# Extended similarity indices: the benefits of comparing more than two objects simultaneously. Part 2: speed, consistency, diversity selection

**DOI:** 10.1186/s13321-021-00504-4

**Published:** 2021-04-23

**Authors:** Ramón Alain Miranda-Quintana, Anita Rácz, Dávid Bajusz, Károly Héberger

**Affiliations:** 1 Department of Chemistry, University of Florida, Gainesville, FL 32603 USA; 2 Plasma Chemistry Research Group, Research Centre for Natural Sciences, Magyar tudósok krt. 2, 1117 Budapest, Hungary; 3 Medicinal Chemistry Research Group, Research Centre for Natural Sciences, Magyar tudósok krt. 2, 1117 Budapest, Hungary

**Keywords:** Multiple comparisons, Computational complexity, Scaling, Rankings, Extended similarity indices, Consistency, Molecular fingerprints, Sum of ranking differences

## Abstract

Despite being a central concept in cheminformatics, molecular similarity has so far been limited to the simultaneous comparison of only two molecules at a time and using one index, generally the Tanimoto coefficent. In a recent contribution we have not only introduced a complete mathematical framework for extended similarity calculations, (i.e. comparisons of more than two molecules at a time) but defined a series of novel idices. Part 1 is a detailed analysis of the effects of various parameters on the similarity values calculated by the extended formulas. Their features were revealed by sum of ranking differences and ANOVA. Here, in addition to characterizing several important aspects of the newly introduced similarity metrics, we will highlight their applicability and utility in real-life scenarios using datasets with popular molecular fingerprints. Remarkably, for large datasets, the use of extended similarity measures provides an unprecedented speed-up over “traditional” pairwise similarity matrix calculations. We also provide illustrative examples of a more direct algorithm based on the extended Tanimoto similarity to select diverse compound sets, resulting in much higher levels of diversity than traditional approaches. We discuss the inner and outer consistency of our indices, which are key in practical applications, showing whether the *n*-ary and binary indices rank the data in the same way. We demonstrate the use of the new *n*-ary similarity metrics on *t*-distributed stochastic neighbor embedding (*t*-SNE) plots of datasets of varying diversity, or corresponding to ligands of different pharmaceutical targets, which show that our indices provide a better measure of set compactness than standard binary measures. We also present a conceptual example of the applicability of our indices in agglomerative hierarchical algorithms. The Python code for calculating the extended similarity metrics is freely available at: https://github.com/ramirandaq/MultipleComparisons

**Supplementary Information:**

The online version contains supplementary material available at 10.1186/s13321-021-00504-4.

## Introduction

Molecular similarity is a key concept in cheminformatics, drug design and related subfields [1, 2]. However, the quantification of molecular similarity is not a trivial task. Generally, binary fingerprints serve to define binary similarity (and distance) coefficients [3], which are routinely used in virtual screening [4], fragment-based de novo ligand design [5–8], hit-to-lead optimization [9], etc.

It is well- known that “the results of similarity assessment vary depending on the compound representation and metric” [10–12]. Willett carried out a detailed comparison of a large number of similarity coefficients and established that the “well-known Tanimoto coefficient remains the method of choice for the computation of fingerprint-based similarity” [13]. He also calculated multiple database rankings using a fixed reference structure and the rank positions were concatenated, in a process called “similarity fusion” [14]. On the other hand, Martin et al. have also called for attention that the “widely and almost exclusively applied Tanimoto similarity coefficient has deficiencies together with the Daylight fingerprints” [15]. If the compounds are selected using an optimal spread design*,* “the Tanimoto coefficient is intrinsically biased toward smaller compounds, when molecules are described by binary vectors with bits corresponding to the presence or absence of structural features” [16].

In our earlier investigations we could prove the equivalency of several coefficients [17], as well as identify a few alternatives to the popular Tanimoto similarity [18]. We have also dedicated a paper to develop an efficient mathematical framework to study the consistency of arbitrary similarity metrics [19]. It is also worth noting that Tanimoto and other metrics can also be applied to quantify field-based representations, like shape similarity [20].

Classically, we can estimate the diversity of a compound set with binary comparisons by calculating its full similarity matrix. Likewise, popular diversity selection algorithms require pre-calculating the full similarity matrix of the compound pool. While this is fine up until a certain size, the similarity matrix calculation scales quadratically with the number of molecules, O(*N*^2^), resulting in very long computation times for larger sets. Methods to speed up these routine calculations are therefore sought after.

To note, one major train of thought for cutting down on computation times began with the introduction of the modal fingerprint [21]. Modal fingerprints are consensus fingerprints that collect the common features of a compound set, which can later be used for comparing sets, or as queries for similarity screening. The concept was further developed by the Medina-Franco group, introducing database fingerprints [22] (DFP) and statistical-based database fingerprints [23] (SB-DFP), with more sophisticated mathematical backgrounds.

By contrast, we have set out to extend the notion of similarity comparisons from two molecules (objects) to many (*n*). In our companion paper, we introduced the full mathematical framework for a series of new similarity indices, which are applicable for multiple (or *n*-ary, as opposed to pairwise) comparisons with and without weighting alike [24]. This is also briefly summarized in the “Extended similarity indices—theory” section of this article.

Our work has some common roots with modal fingerprints and its successors, chiefly in looking for the bit positions that are common to a certain percentage of a compound database (which we term similarity counters here). However, instead of identifying a consensus fingerprint to provide a simplified representation of a large compound set, we use our approach to quantify its overall similarity, extending the concept of similarity from two to many (*n*) molecules. With this, we avoid any information loss that is inherent to modal fingerprints and their successors, while providing a way to quantify compound set similarity with an algorithm that scales as O(*N*).

Here we demonstrate the (i) speed superiority of the extended similarity coefficients *i.e*. how the new indices outperform their binary analogues; (ii) how the new indices are superior in diversity selection; (iii) the robustness of extended coefficients, when changing the coincidence threshold (γ, a continuous meta parameter), and their consistency with the standard binary similarity indices; (iv) the behavior of extended similarity indices as compactness measures on selected datasets; and (v) their utility in hierarchical clustering by providing novel linkage criteria.

## Computational methods

### Extended similarity indices—theory

The companion paper contains the theoretical description and detailed statistical characterization of the extended similarity indices [24]. Nonetheless, to the convenience of the reader, a brief summary is included here.

The extended (or *n*-ary) similarity indices calculate the similarity of a set of an arbitrary number (*n*) of objects (bitstrings, molecular fingerprints), instead of the usual pairwise comparisons. To achieve that, we have extended the existing mathematical framework of similarity metrics. Whereas in binary comparisons, we can count the number of positions with 1–1, 1–0, 0–1, or 0–0 coincidences (usually termed *a*, *b*, *c* and *d*, respectively), in extended comparisons, we have more counters with the general notation ${C}_{n(k)}$, meaning *k* occurrences of “on” (1) bits out of a total of *n* objects. Let us note that *a* and *d* encode features of similarity and *b* and *c* encode features of dissimilarity in pairwise comparisons (although considering 0–0 coincidences or *d* as similarity features is optional, as reflected in the definition of some of the most popular similarity metrics, including the Tanimoto index [17]). By analogy, the key concept of our methodology is to classify the larger number of counters ${C}_{n(k)}$ into similarity and dissimilarity counters with a carefully designed indicator that reflects the a priori expectation for the number of co-occurring 1 bits (coincidence threshold or *γ*). To construct the extended similarity metrics, we simply replace the terms *a*, *b*, *c* and *d* in the definition of binary metrics with the respective sums of 1-similarity (*a*), dissimilarity (*b* + *c*) and, if needed, 0-similarity (*d*) counters. As a result, we will have a single similarity value for our set of *n* objects. Optionally, we can apply a weighting scheme to express the greater contributions to similarity for those counters with a larger number of co-occurrences *k*. To note, all of our metrics are consistent with the “traditional” binary definitions, in that they reproduce the original formulas when *n* = 2. The Python code for calculating the extended similarity metrics is freely available at: https://github.com/ramirandaq/MultipleComparisons

**Fig. 1:**
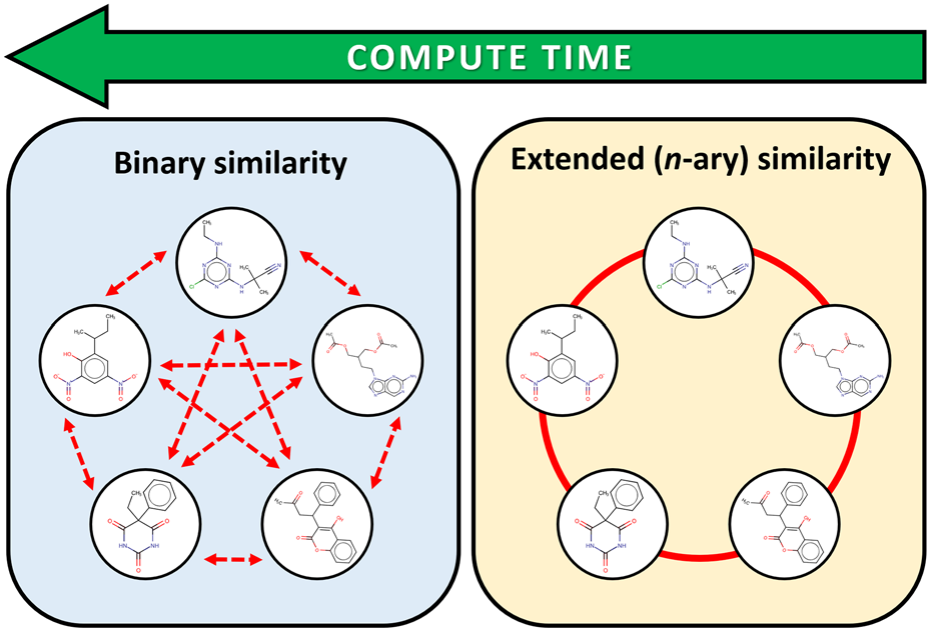
Illustration for the extended similarity metrics versus binary comparisons. A large number of pairwise comparisons is not necessarily able to reveal essential similarities between multiple molecules, despite of the significantly more calculations

Figure 1 is an illustrative visualization of the difference between the binary comparisons and *n*-ary comparisons with the example of five compounds.

### Datasets and fingerprint generation

In order to evaluate our extended similarity metrics in real-life scenarios, we have chosen to generate popular molecular fingerprints for compound sets of various sizes, selected based on different principles—and therefore representing different levels of average similarity. Specifically, molecules were selected from the Mcule database [25] of purchasable compounds (> 33 M compounds in total) either: (i) randomly, (ii) by maximizing their similarity, or (iii) by maximizing their diversity (the latter two were achieved with the LazyPicker algorithm implemented in the RDKit, maximizing the similarity or dissimilarity of the respective sets). A fourth principle for compound set selection was assembling molecule sets, where every molecule shares a common core scaffold. For reasons of practicality, this was achieved by selecting molecules randomly from the ZinClick database: a database of over 16 M 1,2,3-triazoles. [26, 27] To ensure that the small core scaffold (5 heavy atoms) attributes to a significant portion of the molecules, we imposed a constraint that only molecules with at most 15 heavy atoms in total were picked (thus, at least 33% of the basic structures of any two molecules were identical). The resulting sets were termed “random” (R), “similar” (S), “diverse” (D), and “triazole” (T), respectively. Duplicates were removed and from each SMILES entry, only the largest molecule was kept, thereby removing any salts. For each selection principle, compound sets of 10, 100, 1000, 10,000 and 100,000 molecules were generated. The sets were stored as SMILES codes, which were, in turn, used to generate MACCS [2] and Morgan [28] fingerprints, the latter with a radius of 4 and an addressable space (fingerprint length) of either 1024, 2048 or 4096 bits. For the compound set selection and fingerprint generation tasks detailed above, the RDKit cheminformatics toolkit was utilized [29]. In the following sections, we apply our newly introduced extended similarity metrics, and also traditional pairwise similarity calculations to quantify the similarities of the resulting sets and to characterize the behavior of the extended similarity metrics on molecule sets with varying size and overall level of similarity. For the clustering case study, two compound sets were collected from recent works, corresponding to two JAK inhibitor scaffolds (25 indazoles [30] and 7 pyrrolo-pyrimidines [31]). Preparation and fingerprints generation of these sets was carried out as detailed above.

### Visualization of target-specific compound sets

To highlight the applicability of the new extended similarity indices in drug design and computational medicinal chemistry, we have compiled several datasets with ligands of specific, pharmaceutically relevant protein targets. Specifically, 500 randomly selected ligands were picked for two closely related oncotargets, Bruton’s tyrosine kinase (BTK) and Janus kinase 2 (JAK2) and a structurally dissimilar therapeutic target, the β_2_ adrenergic receptor (ligands with an experimental IC_50_/EC_50_/K_d_/K_i_ value of 10 µM or better were picked from the ChEMBL database after duplicate removal and desalting) [32, 33]. Additionally, a larger dataset of cytochrome P450 (CYP) 2C9 ligands (2965 inhibitors with a potency of 10 µM or better and 6046 inactive species) was downloaded from Pubchem Bioassay (AID 1851) [^34^]. Cytochrome P450 (CYP) enzymes are of key importance for drug metabolism and are therefore heavily studied in medicinal chemistry and drug design [35].

In order to visualize the mentioned datasets, we have generated their Morgan fingerprints (radius: 4, length: 1024) and projected the datasets to two dimensions with *t*-distributed stochastic neighbor embedding (*t*-SNE), [36] as implemented in the machine learning package Scikit-learn, [37] with the following settings: perplexity = 30, metric = ‘jaccard’, init = ‘pca’ (initial embedding), n_components = 2.

## Results

### Time analysis

One of the biggest practical advantages of the extended similarity indices is that now we can calculate the overall similarity of a group of molecules much more efficiently than by using the traditional binary comparisons. At a heuristic level, when we have a set with *N* molecules and calculate its chemical diversity using binary comparisons, we first need to select all possible pairs of molecules; then, calculate the similarity of each pair, and finally average the result [38, 39]. There will be $N(N-1)\left/ 2 \right.$ pairs i.e. O(*N*^2^) operations are to be performed. In other words, the time required to calculate the similarity of a set of molecules is expected to grow quadratically with the size of the set. On the other hand, if we use *n*-ary indices, we can compare all of the molecules at the same time, which we expect to scale linearly with the size of the system, that is, in O(*N*).

**Fig. 2:**
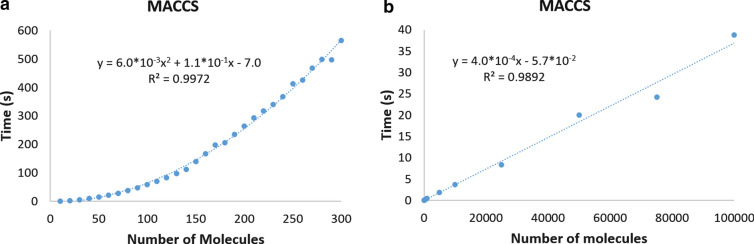
Average time required to calculate the set similarity of the different datasets using MACCS fingerprints with binary (**a**) and *n*-ary (**b**) similarity indices

This can be easily seen in Fig. 2, where we show the different times required to compare datasets using binary or *n*-ary indices when we use MACCS fingerprints (the same trends are observed for the other fingerprint types, as shown in the Additional file 1: Sect. 1). Remarkably, following these trends, estimating the similarity of one million molecules takes 400 s with *n-*ary comparisons, and close to 190 years with binary comparisons.

The speed gain provided by our indices means that we can quantify the similarity of sets with our new indices that are completely inaccessible by current methods, thus allowing us to apply the tools of comparative analysis to the study of more complex databases. This can prove key in the study of chemical diversity [40–42]. The remarkable efficiency of our indices can be exploited in many different scenarios. For instance, the standard way to compare two sets of molecules requires us first to determine the medoid of each set. Traditional algorithms can do this in O(*N*^2^) (if we want to exactly calculate the medoid), or in O($\frac{NlogN}{{\varepsilon }^{2}}$) (if we want to estimate the medoid up to a given error ε). However, with our indices we can just directly compare both sets requiring only O(*N*) operations. We can directly apply our indices in diversity picking, or use them with novel linkage criteria in agglomerative clustering algorithms. We demonstrate the former in the next section, and the latter application in the “Clustering based on extended similarity indices” section.

### Diversity selection

The key advantage of our method in diversity selection is that we can quantify the similarity of a set in O(*N*) while working with the complete representation of the data. One could think of doing this using self-organizing maps [43] (SOMs), or multidimensional scaling [44] based on different molecular descriptors or fingerprint types. However, these alternatives cannot quantify the diversity in an exact way, rather they are realizing a kind of clustering or mapping of the databases and visualize the differences in a heatmap or scatterplot (thus inevitably reducing the complexity of the initial data by representing it in an approximated way). Binary similarity metrics have also been extensively used in the past decades to quantify the overall similarity/diversity of a database, but they are not a viable option for larger databases due to their time-demanding calculation process. In this sense, our method produces a fast, accurate and superior measure of the diversity of a set.

Probably the most popular way to select a diverse set of molecules from a dataset makes use of the MaxMin algorithm: [45, 46].

- If no compounds have been picked so far, choose the 1st picked compound at random.
- Repeatedly, calculate the (binary) similarities between the already picked compounds and the remaining compounds in the dataset (compound pool). Select the molecule from the compound pool that has the smallest value for the biggest similarity between itself and the already selected compounds.
- Continue until the desired number of picked compounds has been selected (or the compound pool has been exhausted).

The MaxSum diversity algorithm [47] is closely related to MaxMin, being also based on traditional binary similarity measures, but differing in the selection step:

- If no compounds have been picked so far, choose the 1st picked compound at random.
- Repeatedly, calculate the (binary) similarities between the already picked compounds and the compound pool. Select the molecule from the pool that has the minimum value for the sum of all the similarities between itself and the already selected compounds.
- Continue until the desired number of picked compounds has been selected (or the compound pool has been exhausted).

Inspired by these methods, here we propose a modified algorithm that directly attempts to maximize the dissimilarity between the selected compounds (we can call this the “Max_nDis” algorithm):

- If no compounds have been picked so far, choose the 1^st^ picked compound at random.
- Repeatedly, given the set of compounds already picked *P*_n_ = $\left\{{M}_{1},{M}_{2},\dots ,{M}_{n}\right\}$ select the compound *M*’ such that the set $\left\{{M}_{1},{M}_{2},\dots ,{M}_{n},{M}^{^{\prime}}\right\}$ has the minimum similarity (as calculated using one of our *n*-ary indices).
- Continue until the desired number of picked compounds has been selected (or the compound pool has been exhausted).

The key difference between these algorithms is a conceptual one: while in MaxMin and MaxSum a new compound is added by maximizing some local (in most cases binary) criterion; in our method, the new compounds are explicitly added by directly maximizing the diversity of the new set. Our method provides a more direct route to obtaining chemically diverse sets, because this is the direct criterion in our optimization. We can compare this conceptual difference to optimization algorithms that locate either a local minimum or the global minimum of the abstract space being investigated (with the latter usually being substantially slower). In this analogy, the Max_nDis algorithm would be similar to an optimization algorithm that locates the global minimum, but with the same speed as a local optimization algorithm (which would correspond to the MaxMin and MaxSum pickers).

**Fig. 3:**
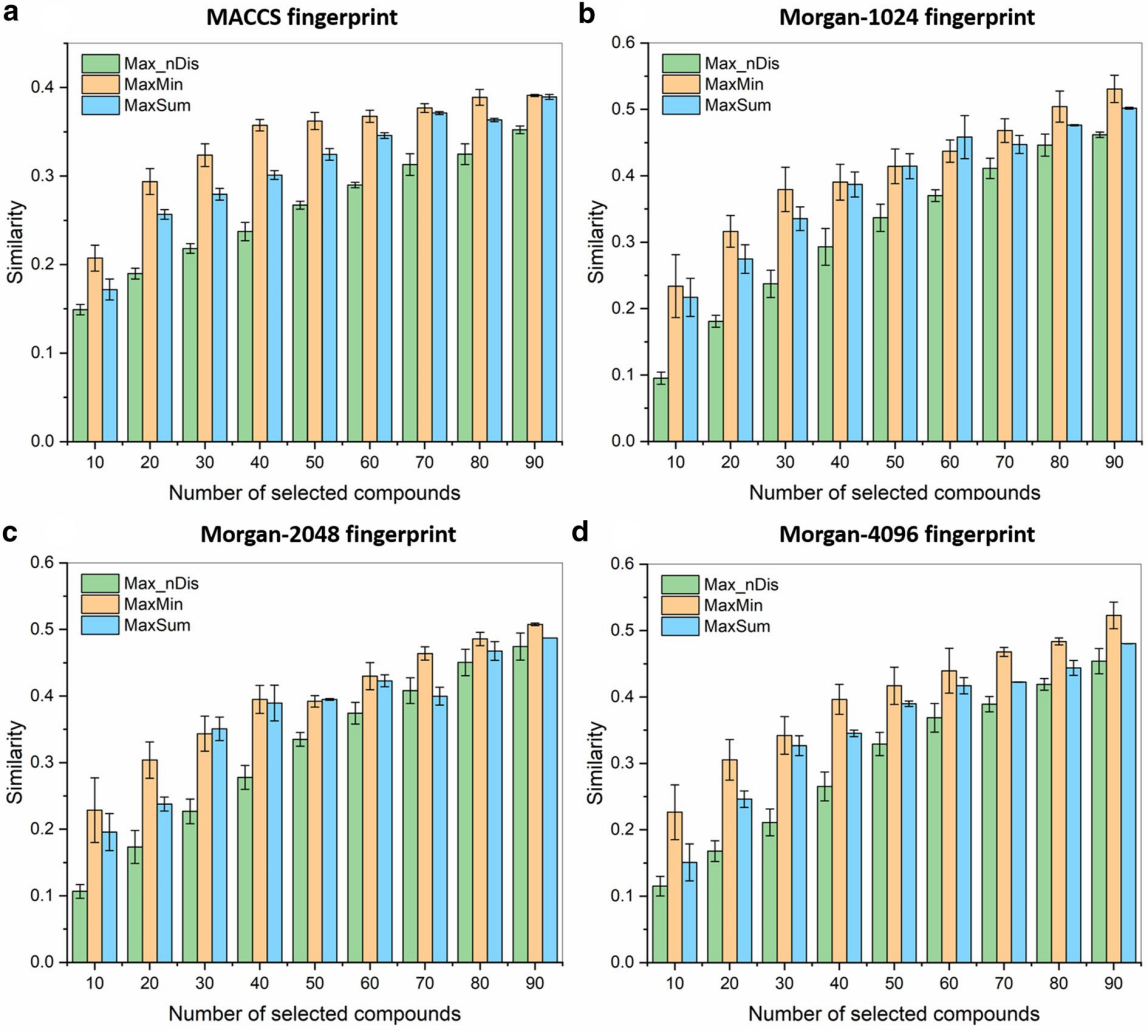
*n*-ary Jaccard-Tanimoto (JT) similarities of diverse sets, selected with the MaxMin (orange), MaxSum (blue), and Max_nDis (green) algorithms. Error bars correspond to standard deviations derived by seven random initialization

To illustrate this, we have compared the MaxMin, MaxSum and Max_nDis algorithms for four types of fingerprints, four datasets with varying levels of similarity, and an additional, larger dataset of cytochrome P450 2C9 inhibitors. In all cases, we ran the algorithms several times (7), so we were able to sample several random initial starting points. We report the average of the similarities obtained these different runs, and also the corresponding standard deviations, which allow us to more clearly distinguish between the different algorithms. In our first test, 10, 20, 30, …, 90 diverse molecules were selected from the “random” (R) compound set of 100 molecules. Figure 3 shows the corresponding results in the case of different fingerprint types (MACCS, Morgan-1024, Morgan-2048 and Morgan-4096). In all cases, and even with a relatively small pool for picking (80–90 selected out of 100), the Max_nDis algorithm selected more diverse sets than MaxMin and MaxSum.

**Fig. 4:**
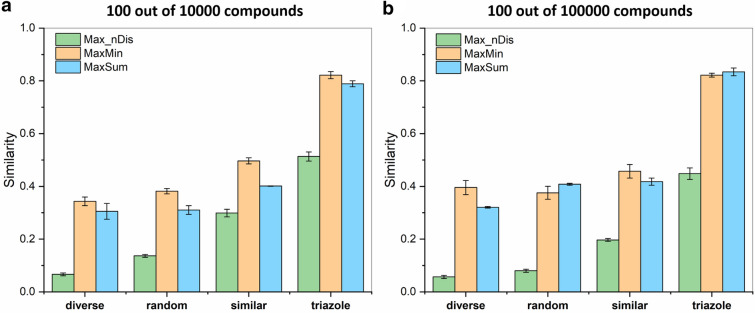
*n*-ary Jaccard-Tanimoto (JT) similarities of diverse sets, selected with the MaxMin (orange), MaxSum (blue), and Max_nDis (green) algorithms. Error bars correspond to standard deviations derived by seven random initialization

In the next step, we have selected 100 molecules from the larger (10,000 and 100,000 molecules) “random” (R), “similar” (S), “diverse” (D), and “triazole” (T) datasets with MaxMin, MaxSum, and our algorithm, as well. Figure 4 shows that Max_nDis was consistently superior to MaxMin and MaxSum. This was particularly outstanding for the datasets that were more diverse to start with (“random” and “diverse”).

**Fig. 5:**
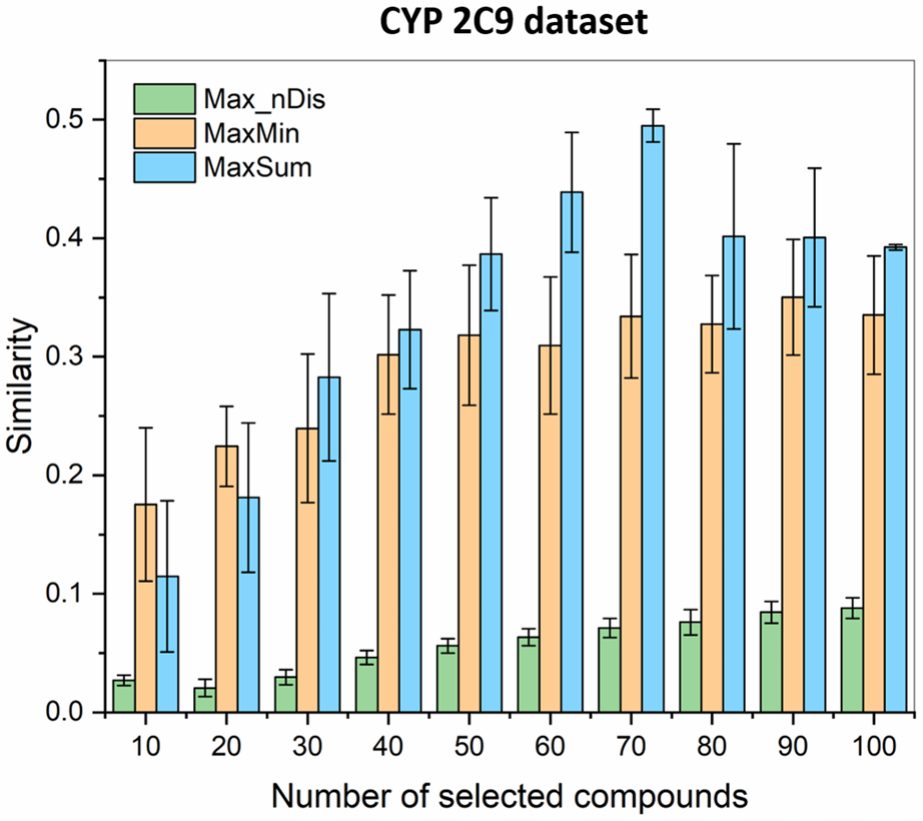
*n*-ary Jaccard-Tanimoto (JT) similarities of diverse sets, selected with the MaxMin (orange), MaxSum (blue), and Max_nDis (green) algorithms. Error bars correspond to standard deviations derived by seven random initialization

Finally, we have compared the selection algorithms for a larger dataset of cytochrome P450 2C9 inhibitors (2965). The results clearly show (Fig. 5), that diversity selection based on the extended similarity metrics was able to produce drastically more diverse sets of 10, 20, 30, …, 100 molecules.

The Max_nDis algorithm has the same time scaling as MaxMin and MaxSum, but routinely resulted in compound sets that are 2–3 times more diverse. The differences were, logically, smaller, when we have selected the molecules from a smaller pool (Fig. 3), but were especially striking for the CYP 2C9 dataset, where the smallest sets (10 and 20 molecules) could be selected with *n*-ary similarities below 0.03, and even for 100 selected compounds, this did not increase to 0.1 (vs*.* close to 0.4 for MaxMin and MaxSum). We can also observe that the overall similarity increases monotonically with the size of the selected set in case of the Max_nDis algorithm (unless the compound pool is nearly exhausted, e.g. > 80 compounds selected from 100, see Fig. 3), which is consistent with the fact that it is used as the direct objective of the picking itself.

### *n*-ary indices: robustness and consistency

A key factor in the applicability of our new indices is their robustness, which we define as their ability to provide consistent results even when we modify some of the parameters used to calculate them, for instance, when we change the coincidence threshold (γ). Let us say that we have two molecular sets, *A* and *B* (both having the same number of elements), and an *n*-ary similarity index ${s}_{n}$. We can measure their set similarity using a given coincidence threshold, γ_1_, which we will denote by: ${s}_{n}^{\left({\gamma }_{1}\right)}\left(A\right)$, ${s}_{n}^{\left({\gamma }_{1}\right)}\left(B\right)$. Without losing any generality we can say that *A* is more similar than *B*, that is: ${s}_{n}^{\left({\gamma }_{1}\right)}\left(A\right)>{s}_{n}^{\left({\gamma }_{1}\right)}\left(B\right)$. Then, the results obtained using index *s*_*n*_ will be robust, inasmuch this relative ranking does not change, if we pick another coincidence threshold, *i.e.* if for ${\gamma }_{2}\ne {\gamma }_{1}$ we also have ${s}_{n}^{\left({\gamma }_{2}\right)}\left(A\right)>{s}_{n}^{\left({\gamma }_{2}\right)}\left(B\right)$. Notice that we can write this property as:

1 $$\left[{s}_{n}^{\left({\gamma }_{1}\right)}\left(A\right)-{s}_{n}^{\left({\gamma }_{1}\right)}\left(B\right)\right]\left[{s}_{n}^{\left({\gamma }_{2}\right)}\left(A\right)-{s}_{n}^{\left({\gamma }_{2}\right)}\left(B\right)\right]>0$$

This is highly reminiscent of the consistency relationship for comparative indices [48, 49], and for this reason, from now on we will refer to this property as internal consistency.

**Fig. 6:**
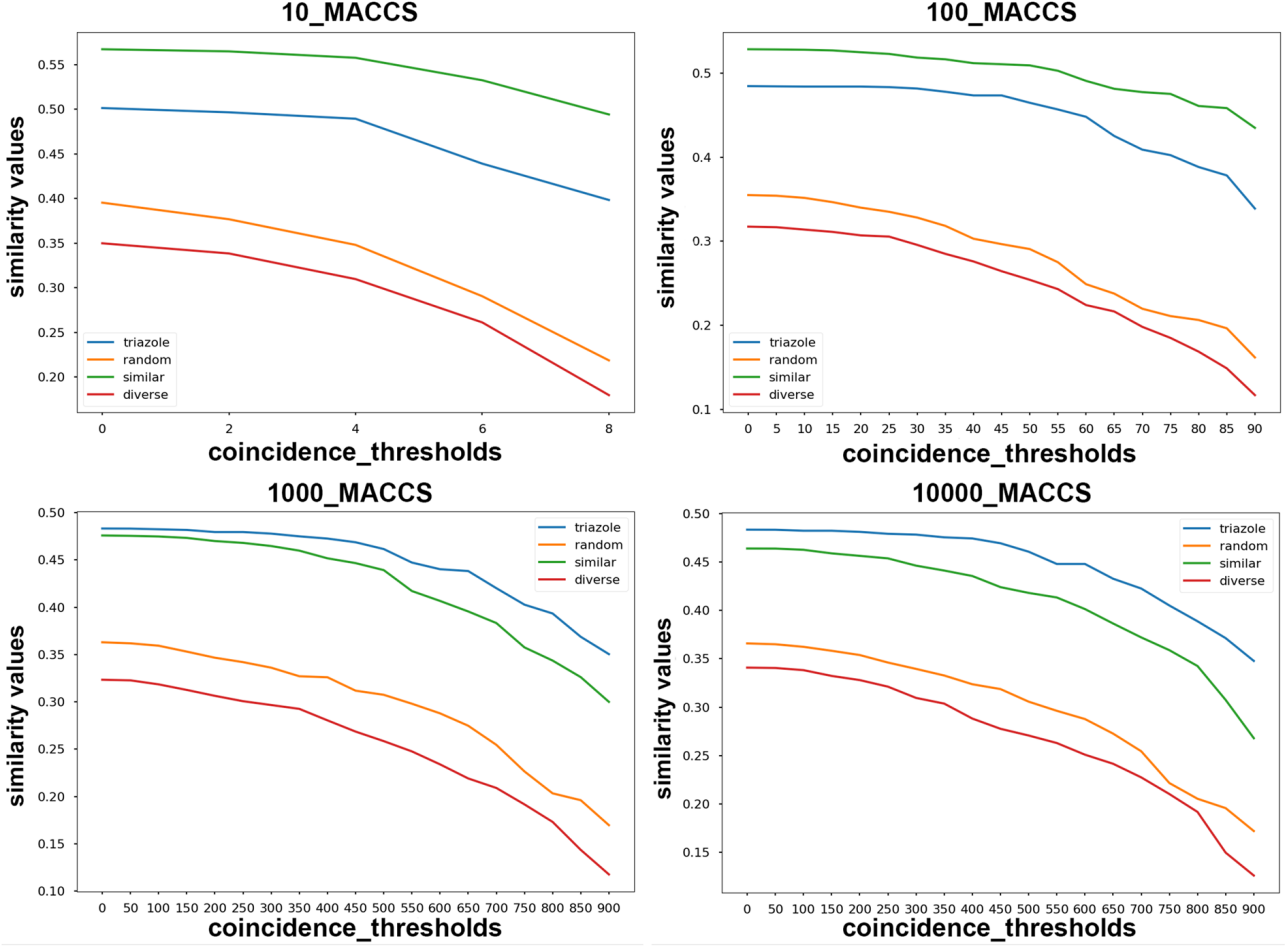
Set similarity calculated with the eFainw index for the different datasets and sizes considered using MACSS fingerprints. The abbreviations are resolved in the Appendix 1 and also in ref. [24]

In order to study the internal consistency of the extended indices, we focused on the similar (S) and triazole (T) datasets with 10, 100, 1000, and 10,000 molecules. In Fig. 6 we show an example of the non-weighted extended Faith (eFai) index (eFainw) using the MACSS fingerprints for different set sizes. We see that the T (blue) and S (green) lines never cross each other, which means that the relative rankings of these sets is preserved (in other words, this index is internally consistent under the present conditions for the sets T and S).

A more quantitative measure of this indicator can be obtained by calculating the fraction of times that the relative rankings of the S and T sets were preserved. This simple measure (which we call the *internal consistency fraction*, ICF) allows us to quickly quantify the internal consistency of an index since we can readily identify a greater value with a greater degree of internal consistency (a value of 1 corresponds to a perfectly internally consistent index, as it was the case for the eFainw index shown in Fig. 6). The detailed results are presented in the Additional file 1: Section 2. It is reassuring to notice that many of the indices identified as best in the accompanying paper (like the eBUBnw and eFainw indices) provide the highest ICF values.

Another important measure of robustness is the consistency of the extended similarity metrics with the corresponding standard binary similarity indices. Given an *n*-ary index calculated with a coincidence threshold γ, ${s}_{n}^{\left(\gamma \right)}$, and a binary index ${s}_{2}$, they will be consistent if for any two sets *A*, *B* we have:

2 $$\left[{s}_{n}^{\left(\gamma \right)}\left(A\right)-{s}_{n}^{\left(\gamma \right)}\left(B\right)\right]\left[{s}_{2}\left(A\right)-{s}_{2}\left(B\right)\right]>0$$

To avoid confusion with the previously introduced internal consistency, we will refer to Eq. (2) as the external consistency. It is obvious that the external consistency indicates whether the *n*-ary and binary indices rank the data in the same way. It is thus natural to use sum of ranking differences (SRD) to analyze this property. Briefly, SRD is a statistically robust comparative method based on quantifying the Manhattan distances of the compared data vectors from an ideal reference, after rank transformation (a more detailed description of the method is included in the accompanying paper). If the reference in the SRD analysis is selected to be the binary results, then the indices will be externally consistent if and only if SRD = 0.

**Fig. 7:**
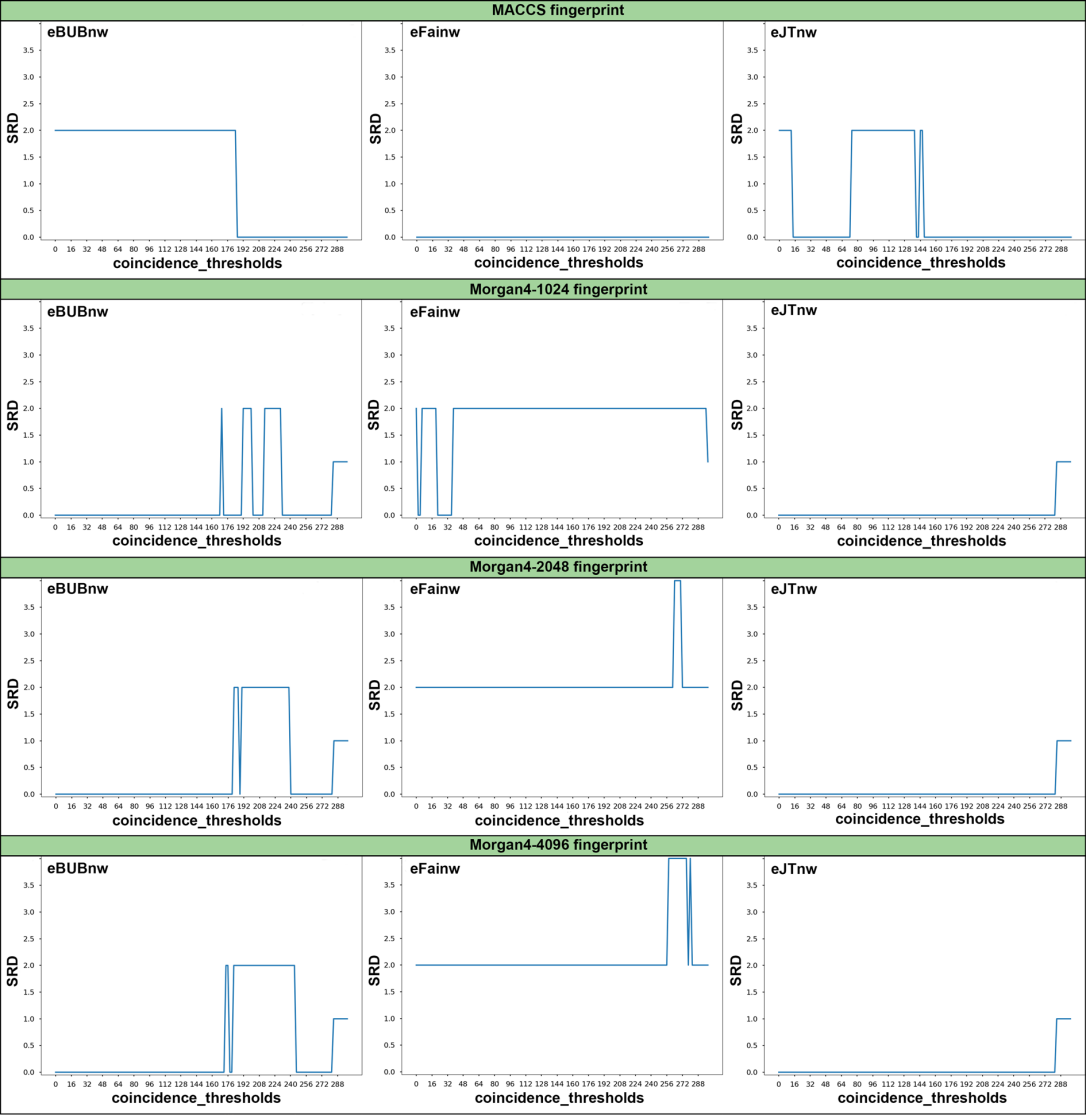
SRD variation with the coincidence threshold for the eBUBnw, eFainw, and eJTnw indices over sets with 300 molecules for the MACSS, Morgan4_1024, Morgan4_2048, and Morgan4_4096 fingerprints

In Fig. 7 we show how the SRD changes for several indices when we vary the coincidence threshold. We selected sets with 300 molecules to allow us to explore a large number of coincidence thresholds. As it was the case for the internal consistency (Additional file 1: Table S1), here we see once again that the choice of fingerprint greatly impacts the consistency. Remarkably, the eJTnw index is particularly well-behaved if we use Morgan4 fingerprints, being externally consistent for the vast majority (142 out of 150) of the coincidence thresholds analyzed. This is reassuring, given the widespread use of the Jaccard-Tanimoto index [13, 16, 17].

Analogously to the ICF, we can define an external consistency fraction, ECF for measuring the fraction of times that the SRD is zero for all the coincidence thresholds that could be analyzed for a given set of molecules. In other words, the ECF is an indication of how often the *n*-ary index ranks the data in exactly the same order as the binary indices (ECF values are summarized in Table S2). Once again it is comforting to see that many of the best indices with respect to our previous SRD and ICF analyses are also the best with respect to the ECF. The detailed results on external consistency are presented in the Additional file 1: Section 3, along with SRD-based comparisons of the consistency measures according to several factors, such as the applied fingerprints and the effect of weighting (Additional file 1: Section 4).

### Extended similarity indices on selected datasets

**Fig. 8:**
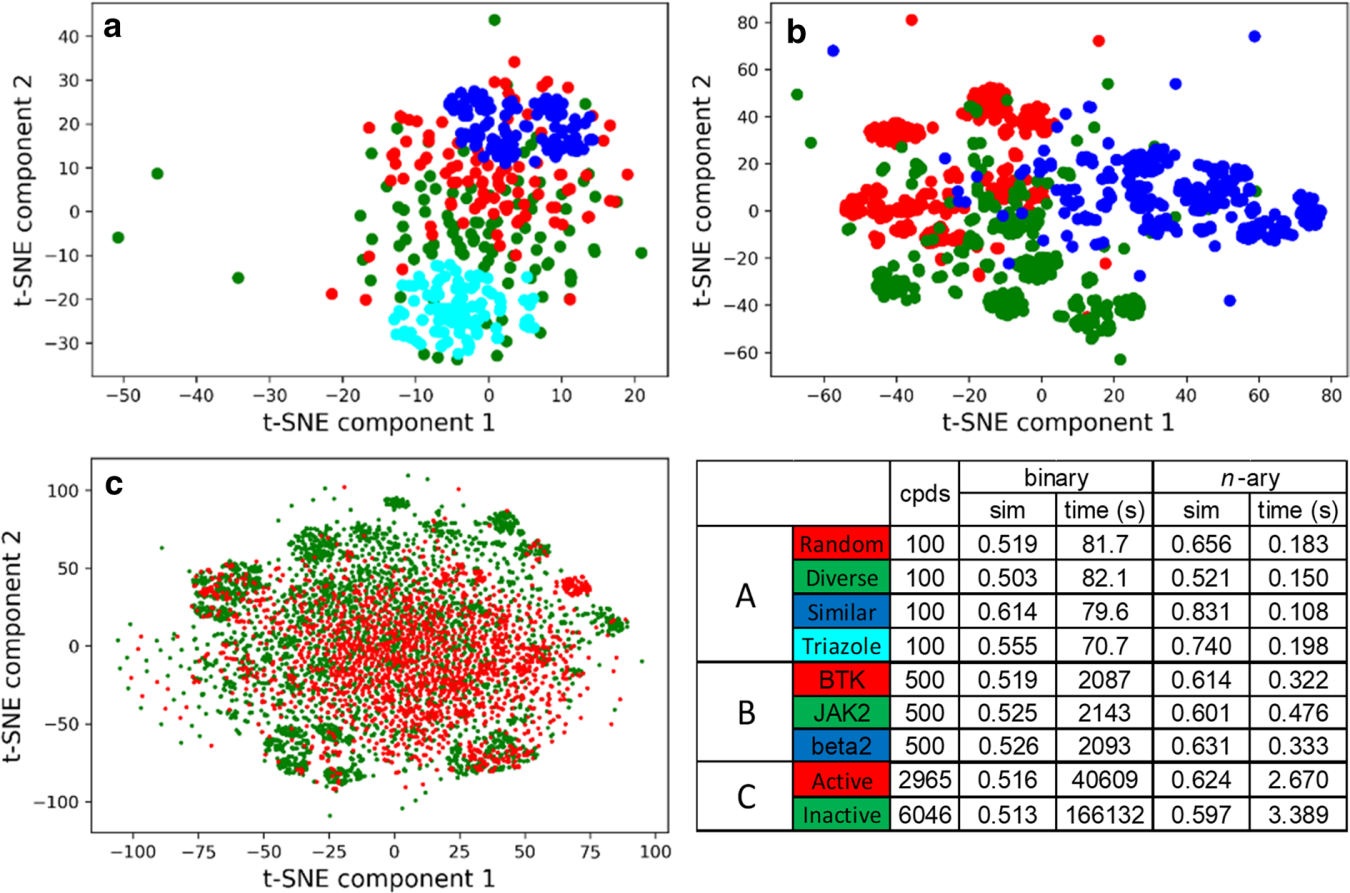
*t*-distributed stochastic neighbor embedding (*t*-SNE) of: (**a**) the sets of 100 compounds selected with the different selection methods, (**b**) sets of 500 ligands of different pharmaceutical targets, and (**c**) sets of cytochrome P450 2C9 ligands and inactive compounds from PubChem Bioassay 1851. The table summarizes the number of compounds in the sets, as well as computation times and average similarities (averaged over the 19 non-weighted similarity metrics, and, for the binary comparisons, also over all possible compound pairs)

Our indices can also be used to analyze several datasets, for instance: the 100-compound selections from the commercial libraries (random, diverse, similar, triazole, see  "Datasets and fingerprint generation" section), as well as 500 randomly selected ligands for three therapeutical targets, and a larger dataset (9011 compounds) from the PubChem Bioassay dataset AID 1851, containing cytochrome P450 2C9 enzyme inhibitors and inactive compounds. We have applied *t*-distributed stochastic neighbor embedding (*t*-SNE) to visualize the sets in 2D (Fig. 7) and compiled the runtimes and average similarity values calculated with the binary and the non-weighted extended similarity metrics (where *n* was the total number of compounds, *i.e.* all compounds were compared simultaneously). The *t*-SNE plots were generated from Morgan fingerprints (1024-bit) and are provided solely to illustrate the conclusions detailed here. The three case studies correspond to distinct scenarios. For the commercial compounds, the sets selected by maximizing similarity, or fixing the core scaffold (triazole) clearly form more compact groups than the randomly picked compounds or the diverse set (Fig. 8a). The BTK and JAK2 inhibitors, and the β_2_ adrenergic receptor ligands form groups of similar compactness, with moderate overlap (Fig. 8b). The CYP 2C9 enzyme inhibitors and inactive compounds form loose and completely overlapping groups (Fig. 8c).

The key results are summarized in the table in Fig. 8. This lists the *n*-ary similarities (averaged over 19 non-weighted *n*-ary similarity metrics) and the corresponding binary similarities (averaged over 19 non-weighted binary similarity metrics and over all pairs of compounds). We also present the computation times for all of the clusters in the *t*-SNE plots, so that the reader can match the quantitative information against the visual representation of the clusters. We wanted to highlight here the utility of the new *n*-ary metrics to quantify the overall similarity (or conversely, diversity) of compound sets. First, it is clear that the extended similarity metrics offer a tremendous performance gain, with total computation times as low as 2–3 s even for the largest dataset (9011 compounds). By contrast, computation times for the full binary distance matrices range from 1.2 min (100 compounds), to 34–36 min (500 compounds), and to 46 h (6046 compounds). Additionally, it is worth noting that the extended metrics offer a greater level of distinction in terms of the compactness of the sets, ranging from 0.521 (diverse set) to 0.831 (similar set) in the most illustrative case, compared to a range from 0.503 (diverse) to 0.614 (similar) for binary comparisons. While there is almost no distinction in the binary case between the BTK, JAK2 and β_2_ sets, a minimal distinction is still retained by the extended metrics (returning a noticeably higher similarity score for the slightly more compact group of β_2_ ligands). The same observation goes for the CYP 2C9 dataset, where the slightly greater coherence of the group of 2C9 inhibitors is reflected at the level of the second decimal place in the *n-*ary comparisons, but only third decimal place for the “traditional” binary comparisons. Moreover, for the binary calculations of the 2C9 inactive set (6046 compounds), a computer with 64 GB RAM was required to avoid running out of memory and even then, the calculation took almost 2 days to complete (this is contrasted to 3 s of runtime on a more modest machine for the *n-*ary comparisons). In summary, our indices are much better equipped to uncover the relations between the elements of large sets because they take into account all the features of all the molecules at the same time (while scaling much better than traditional binary comparisons).

### Clustering based on extended similarity indices

The success of our indices in quantifying the degree of compactness of a set suggests that they can be also applied in clustering. Traditionally, the similarity or dissimilarity between clusters is given as a function based on binary distance metrics (i.e. reversed similarity), which are then used in a linkage criterion to decide which clusters (or singletons) should be merged in each iteration. The *n*-ary indices, on the other hand, provide an alternative route towards hierarchical agglomerative clustering: we measure the distance (or similarity) between two sets *A* and *B* by forming the set $C=A\cup B$, and then calculating the similarity of all the elements of *C* using an *n*-ary index. The rest of the algorithm proceeds as usual, that is, combining at each step those clusters that are more similar to (or less distant from) each other. In this approach, the *n*-ary similarities effectively act as novel linkage criteria. To showcase the applicability of the new extended similarity metrics in clustering, we have implemented this new agglomerative clustering algorithm based on the extended Jaccard-Tanimoto index (eJT).

**Fig. 9:**
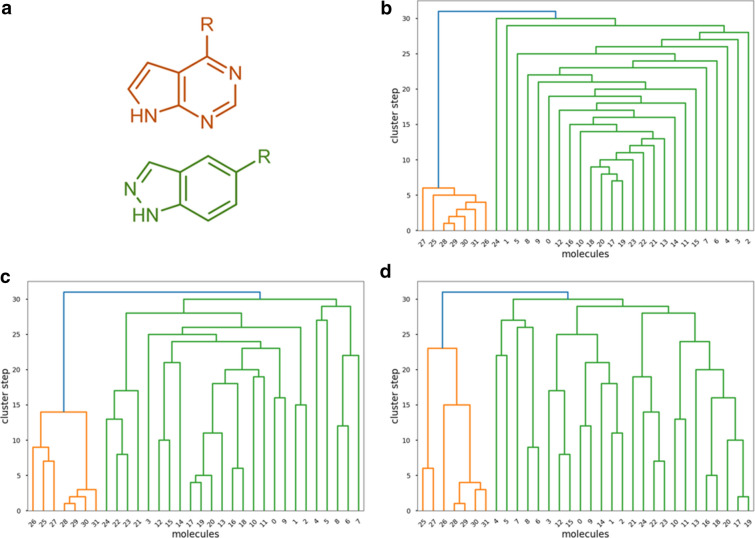
**a** The two core scaffolds of the JAK inhibitor dataset: pyrrolo-pyrimidine (orange) and indazole (green). **b**–**d** Results of agglomerative clustering with the *n-*ary Jaccard-Tanimoto metric (**b**), and the binary JT metric with single linkage (**c**) and binary JT metric with complete linkage (**d**)

For illustrative purposes, we have collected two compound sets from recent works, corresponding to two distinct JAK inhibitor scaffolds (25 indazoles [30] and 7 pyrrolo-pyrimidines [31]). Figure 9 summarizes the results obtained by two “classical” clustering approaches (based on pairwise Tanimoto distances and the single and complete linkage rules), as well as the *n-*ary agglomerative clustering algorithm. It is clear that all three algorithms can distinguish between the two core scaffolds. Additionally, the comparison nicely highlights the difference in the train of thought for the *n*-ary similarity metrics: while classical agglomerative clustering approaches operate with pairwise linkages of smaller subclusters, the *n-*ary algorithm “builds up” the larger, coherent clusters step by step, thereby providing a more compact visual representation for the larger groups. In other words, the *n*-ary indices allow us to analyze the data from a different perspective, thus facilitating to uncover other relations between the objects being studied. It is important to remark that this is merely a proof-of-principle example of the application of our indices to the clustering problem. Uncovering the general characteristics of *n-*ary clustering and further ideas for algorithms need to be further explored in more detail (we are currently working on this direction and the corresponding results will be presented elsewhere).

## Conclusions and summary

In the companion paper, we have introduced a full mathematical framework for extended similarity metrics, i.e. for quantifying the similarities of an arbitrary number (*n*) of molecular fingerprints (or other bitvector-like data structures). Here, after briefly reiterating the core ideas, we show the practical advantages and some prospective applications for the new similarity indices.

First, the calculation of extended similarity indices is drastically faster (more efficient) than the traditional binary indices used so far, scaling linearly with the number of compared molecules, as opposed to the quadratic scaling of calculating full similarity matrices with binary comparisons. To note, calculating the *n*-ary similarity of a set of ~ 6000 compounds took three seconds on a standard laptop, while calculating the binary similarity matrix for the same set took almost two days on a high-end computer.

An important prospective application for the new similarity indices is diversity picking. Here, our Max_nDis algorithm based on the extended Tanimoto index consistently selected much more diverse sets of molecules than currently used algorithms. The reason for this is that the Max_nDis algorithm directly maximizes the diversity (minimizes the *n-*ary similarity) of the selected dataset at each step, while traditional approaches like the MaxMin and MaxSum algorithms individually evaluate the similarities of the next picked compound to the members of the already picked set. It is noteworthy that this result is achieved without increasing the computational demand of the process.

Clustering, as another prospective field of application, showcases the different train of thought behind the agglomerative clustering algorithm we implemented based on the extended Tanimoto similarity, “building up” the larger, more coherent clusters step by step, rather than linking/merging smaller subclusters. Here, implications for further variations of clustering algorithms are wide, and we plan to extend upon this work in the close future.

Further on, we have demonstrated several important features of the new metrics: they are robust or “internally consistent” for different coincidence threshold settings. On the other hand, not all of them are consistent with their binary counterparts in terms of how they rank different datasets (external consistency); this is also influenced by the fingerprint used. Based on these results, a subset of the metrics can be preferred (this includes the extended Jaccard-Tanimoto index), this is detailed in the Supplementary Information. We have also provided visual examples that showcase the capacity of the new indices to distinguish between compact and more diffuse clusters of molecules.

The extended similarity indices provide a new dimension to the comparative analysis, giving us great flexibility at the time of comparing groups of molecules. Now, in this contribution we have shown that these indices are not only attractive from a theoretical point of view, but extremely convenient in practice. This combination of flexibility and unprecedented computational performance is extremely appealing and will allow us to explore the chemical space in novel, more efficient ways.

### Supplementary Information

**Additional file 1**: **Figure S1: **Average time required to calculate the set similarity of the different datasets using Morgan4 fingerprints with *binary* similarity indices. **Figure S2:** Average time required to calculate the set similarity of the different datasets using Morgan4 fingerprints with *n-ary* similarity indices. **Table S1:** Average internal consistency fractions over sets with 10, 20, …, 300 molecules of all the extended similarity indices for all fingerprint types. **Table S2:** Average external consistency fractions over sets with 10, 20,…, 300 molecules of all the extended similarity indices for all fingerprint types. **Figure S3:** SRD analysis for the internal (i) and external (e) consistencies over the different fingerprint types. **Figure S4:** Effect of internal (i) and external consistency (e) on the extended multiple similarity indices. Notation can be found in Appendix 1, and also in the accompanying paper.^4^. **Figure S5:** Effect of weighting on the extended multiple similarity indices. **Figure S6:** Joint effect of internal and external consistency as well as weighting on the extended multiple similarity indices.

## Data Availability

Python code for calculating the extended similarity metrics is freely available at: https://github.com/ramirandaq/MultipleComparisons

## Appendix 1

Extended *n*-ary similarity indices.

**Table:**

Additive indices				
Label	Type	Notation	Name	Equation
eAC	eAC_1	eACw	Extended Austin-Colwell	${s}_{eAC\left(1s\_wd\right)}=\frac{2}{\pi }\mathrm{arcsin}\sqrt{\frac{\sum_{1-s}{f}_{s}\left({\Delta }_{n(k)}\right){C}_{n(k)}+\sum_{0-s}{f}_{s}\left({\Delta }_{n(k)}\right){C}_{n(k)}}{\sum_{s}{f}_{s}\left({\Delta }_{n(k)}\right){C}_{n(k)}+\sum_{d}{f}_{d}\left({\Delta }_{n(k)}\right){C}_{n(k)}}}$
		eACnw		${s}_{eAC\left(1s\_d\right)}=\frac{2}{\pi }\mathrm{arcsin}\sqrt{\frac{\sum_{1-s}{f}_{s}\left({\Delta }_{n(k)}\right){C}_{n(k)}+\sum_{0-s}{f}_{s}\left({\Delta }_{n(k)}\right){C}_{n(k)}}{\sum_{s}{C}_{n(k)}+\sum_{d}{C}_{n(k)}}}$
eBUB	eBUB_1	eBUBw	Extended Baroni-Urbani-Buser	$${s}_{eBUB\left(1s\_wd\right)}=\frac{\sqrt{\left[\sum_{1-s}{f}_{s}\left({\Delta }_{n(k)}\right){C}_{n(k)}\right]\left[\sum_{0-s}{f}_{s}\left({\Delta }_{n(k)}\right){C}_{n(k)}\right]}+\sum_{1-s}{f}_{s}\left({\Delta }_{n(k)}\right){C}_{n(k)}}{\left\{\begin{array}{c}\sqrt{\left[\sum_{1-s}{f}_{s}\left({\Delta }_{n\left(k\right)}\right){C}_{n\left(k\right)}\right]\left[\sum_{0-s}{f}_{s}\left({\Delta }_{n\left(k\right)}\right){C}_{n\left(k\right)}\right]}+\\ \sum_{1-s}{f}_{s}\left({\Delta }_{n\left(k\right)}\right){C}_{n\left(k\right)}+\sum_{d}{f}_{d}\left({\Delta }_{n\left(k\right)}\right){C}_{n\left(k\right)}\end{array}\right\}}$$
		eBUBnw		${s}_{eBUB\left(1s\_d\right)}=\frac{\sqrt{\left[\sum_{1-s}{f}_{s}\left({\Delta }_{n(k)}\right){C}_{n(k)}\right]\left[\sum_{0-s}{f}_{s}\left({\Delta }_{n(k)}\right){C}_{n(k)}\right]}+\sum_{1-s}{f}_{s}\left({\Delta }_{n(k)}\right){C}_{n(k)}}{\left\{\sqrt{\left[\sum_{1-s}{C}_{n\left(k\right)}\right]\left[\sum_{0-s}{C}_{n\left(k\right)}\right]}+\sum_{1-s}{C}_{n\left(k\right)}+\sum_{d}{C}_{n\left(k\right)}\right\}}$
eCT1	eCT1_1	eCT1w	Extended Consonni-Todeschini (1)	${s}_{eCT1\left(1s\_wd\right)}=\frac{\mathrm{ln}\left(1+\sum_{1-s}{f}_{s}\left({\Delta }_{n(k)}\right){C}_{n(k)}+\sum_{0-s}{f}_{s}\left({\Delta }_{n(k)}\right){C}_{n(k)}\right)}{\mathrm{ln}\left(1+\sum_{s}{f}_{s}\left({\Delta }_{n(k)}\right){C}_{n(k)}+\sum_{d}{f}_{d}\left({\Delta }_{n(k)}\right){C}_{n(k)}\right)}$
		eCT1nw		${s}_{eCT1\left(1s\_d\right)}=\frac{\mathrm{ln}\left(1+\sum_{1-s}{f}_{s}\left({\Delta }_{n(k)}\right){C}_{n(k)}+\sum_{0-s}{f}_{s}\left({\Delta }_{n(k)}\right){C}_{n(k)}\right)}{\mathrm{ln}\left(1+\sum_{s}{C}_{n(k)}+\sum_{d}{C}_{n(k)}\right)}$
eCT2	eCT2_1	eCT2w	Extended Consonni-Todeschini (2)	${s}_{eCT2\left(1s\_wd\right)}=\frac{\mathrm{ln}\left(1+\sum_{s}{f}_{s}\left({\Delta }_{n(k)}\right){C}_{n(k)}+\sum_{d}{f}_{d}\left({\Delta }_{n(k)}\right){C}_{n(k)}\right)-\mathrm{ln}\left(1+\sum_{d}{f}_{d}\left({\Delta }_{n(k)}\right){C}_{n(k)}\right)}{\mathrm{ln}\left(1+\sum_{s}{f}_{s}\left({\Delta }_{n(k)}\right){C}_{n(k)}+\sum_{d}{f}_{d}\left({\Delta }_{n(k)}\right){C}_{n(k)}\right)}$
		eCT2nw		${s}_{eCT2\left(1s\_d\right)}=\frac{\mathrm{ln}\left(1+\sum_{s}{f}_{s}\left({\Delta }_{n(k)}\right){C}_{n(k)}+\sum_{d}{f}_{d}\left({\Delta }_{n(k)}\right){C}_{n(k)}\right)-\mathrm{ln}\left(1+\sum_{d}{f}_{d}\left({\Delta }_{n(k)}\right){C}_{n(k)}\right)}{\mathrm{ln}\left(1+\sum_{s}{C}_{n(k)}+\sum_{d}{C}_{n(k)}\right)}$
eFai	eFai_1	eFaiw	extended Faith	${s}_{eFai\left(1s\_wd\right)}=\frac{\sum_{1-s}{f}_{s}\left({\Delta }_{n(k)}\right){C}_{n(k)}+0.5\sum_{0-s}{f}_{s}\left({\Delta }_{n(k)}\right){C}_{n(k)}}{\sum_{s}{f}_{s}\left({\Delta }_{n(k)}\right){C}_{n(k)}+\sum_{d}{f}_{d}\left({\Delta }_{n(k)}\right){C}_{n(k)}}$
		eFainw		${s}_{eFai\left(1s\_d\right)}=\frac{\sum_{1-s}{f}_{s}\left({\Delta }_{n(k)}\right){C}_{n(k)}+0.5\sum_{0-s}{f}_{s}\left({\Delta }_{n(k)}\right){C}_{n(k)}}{\sum_{s}{C}_{n(k)}+\sum_{d}{C}_{n(k)}}$
eGK	eGK_1	eGKw	Extended Goodman–Kruskal	${s}_{eGK\left(1s\_wd\right)}=\frac{2\mathrm{min}\left(\sum_{1-s}{f}_{s}\left({\Delta }_{n(k)}\right){C}_{n(k)},\sum_{0-s}{f}_{s}\left({\Delta }_{n(k)}\right){C}_{n(k)}\right)-\sum_{d}{f}_{d}\left({\Delta }_{n(k)}\right){C}_{n(k)}}{2\mathrm{min}\left(\sum_{1-s}{f}_{s}\left({\Delta }_{n(k)}\right){C}_{n(k)},\sum_{0-s}{f}_{s}\left({\Delta }_{n(k)}\right){C}_{n(k)}\right)+\sum_{d}{f}_{d}\left({\Delta }_{n(k)}\right){C}_{n(k)}}$
		eGKnw		${s}_{eGK\left(1s\_d\right)}=\frac{2\mathrm{min}\left(\sum_{1-s}{f}_{s}\left({\Delta }_{n(k)}\right){C}_{n(k)},\sum_{0-s}{f}_{s}\left({\Delta }_{n(k)}\right){C}_{n(k)}\right)-\sum_{d}{f}_{d}\left({\Delta }_{n(k)}\right){C}_{n(k)}}{2\mathrm{min}\left(\sum_{1-s}{C}_{n(k)},\sum_{0-s}{C}_{n(k)}\right)+\sum_{d}{C}_{n(k)}}$
eHD	eHD_1	eHDw	Extended Hawkins-Dotson	$${s}_{eHD\left(1s\_wd\right)}=\frac{1}{2}\left(\begin{array}{c}\frac{\sum_{1-s}{f}_{s}\left({\Delta }_{n(k)}\right){C}_{n(k)}}{\sum_{1-s}{f}_{s}\left({\Delta }_{n(k)}\right){C}_{n(k)}+\sum_{d}{f}_{d}\left({\Delta }_{n(k)}\right){C}_{n(k)}}+\\ \frac{\sum_{0-s}{f}_{s}\left({\Delta }_{n(k)}\right){C}_{n(k)}}{\sum_{0-s}{f}_{s}\left({\Delta }_{n(k)}\right){C}_{n(k)}+\sum_{d}{f}_{d}\left({\Delta }_{n(k)}\right){C}_{n(k)}}\end{array}\right)$$
		eHDnw		$${s}_{eHD\left(1s\_d\right)}=\frac{1}{2}\left(\begin{array}{c}\frac{\sum_{1-s}{f}_{s}\left({\Delta }_{n(k)}\right){C}_{n(k)}}{\sum_{1-s}{C}_{n(k)}+\sum_{d}{C}_{n(k)}}+\\ \frac{\sum_{0-s}{f}_{s}\left({\Delta }_{n(k)}\right){C}_{n(k)}}{\sum_{0-s}{C}_{n(k)}+\sum_{d}{C}_{n(k)}}\end{array}\right)$$
eRT	eRT_1	eRTw	Extended Rogers-Tanimoto	${s}_{eRT\left(1s\_wd\right)}=\frac{\sum_{s}{f}_{s}\left({\Delta }_{n(k)}\right){C}_{n(k)}}{\sum_{s}{f}_{s}\left({\Delta }_{n(k)}\right){C}_{n(k)}+2\sum_{d}{f}_{d}\left({\Delta }_{n(k)}\right){C}_{n(k)}}$
		eRTnw		${s}_{eRT\left(1s\_d\right)}=\frac{\sum_{s}{f}_{s}\left({\Delta }_{n(k)}\right){C}_{n(k)}}{\sum_{s}{C}_{n(k)}+2\sum_{d}{C}_{n(k)}}$
eRG	eRG_1	eRGw	Extended Rogot-Goldberg	$${s}_{eRT\left(1s\_wd\right)}=\begin{array}{c}\frac{\sum_{1-s}{f}_{s}\left({\Delta }_{n(k)}\right){C}_{n(k)}}{2\sum_{1-s}{f}_{s}\left({\Delta }_{n(k)}\right){C}_{n(k)}+\sum_{d}{f}_{d}\left({\Delta }_{n(k)}\right){C}_{n(k)}}+\\ \frac{\sum_{0-s}{f}_{s}\left({\Delta }_{n(k)}\right){C}_{n(k)}}{2\sum_{0-s}{f}_{s}\left({\Delta }_{n(k)}\right){C}_{n(k)}+\sum_{d}{f}_{d}\left({\Delta }_{n(k)}\right){C}_{n(k)}}\end{array}$$
		eRGnw		${s}_{eRT\left(1s\_d\right)}=\frac{\sum_{1-s}{f}_{s}\left({\Delta }_{n(k)}\right){C}_{n(k)}}{2\sum_{1-s}{C}_{n(k)}+\sum_{d}{C}_{n(k)}}+\frac{\sum_{0-s}{f}_{s}\left({\Delta }_{n(k)}\right){C}_{n(k)}}{2\sum_{0-s}{C}_{n(k)}+\sum_{d}{C}_{n(k)}}$
eSM	eSM_1	eSMw	Extended Simple matching, Sokal-Michener	${s}_{eSM\left(1s\_wd\right)}=\frac{\sum_{s}{f}_{s}\left({\Delta }_{n(k)}\right){C}_{n(k)}}{\sum_{s}{f}_{s}\left({\Delta }_{n(k)}\right){C}_{n(k)}+\sum_{d}{f}_{d}\left({\Delta }_{n(k)}\right){C}_{n(k)}}$
		eSMnw		${s}_{eSM\left(1s\_d\right)}=\frac{\sum_{s}{f}_{s}\left({\Delta }_{n(k)}\right){C}_{n(k)}}{\sum_{s}{C}_{n(k)}+\sum_{d}{C}_{n(k)}}$
eSS2	eSS2_1	eSS2w	Extended Sokal-Sneath (2)	${s}_{eSS2\left(1s\_wd\right)}=\frac{2\sum_{s}{f}_{s}\left({\Delta }_{n(k)}\right){C}_{n(k)}}{2\sum_{s}{f}_{s}\left({\Delta }_{n(k)}\right){C}_{n(k)}+\sum_{d}{f}_{d}\left({\Delta }_{n(k)}\right){C}_{n(k)}}$
		eSS2nw		${s}_{eSS2\left(1s\_wd\right)}=\frac{2\sum_{s}{f}_{s}\left({\Delta }_{n(k)}\right){C}_{n(k)}}{2\sum_{s}{C}_{n(k)}+\sum_{d}{C}_{n(k)}}$
Asymmetric indices				
Label	Type		Name	Equation
eCT3	eCT3_1	eCT3w	Extended Consonni-Todeschini (3)	${s}_{eCT3\left(1s\_wd\right)}=\frac{\mathrm{ln}\left(1+\sum_{1-s}{f}_{s}\left({\Delta }_{n(k)}\right){C}_{n(k)}\right)}{\mathrm{ln}\left(1+\sum_{s}{f}_{s}\left({\Delta }_{n(k)}\right){C}_{n(k)}+\sum_{d}{f}_{d}\left({\Delta }_{n(k)}\right){C}_{n(k)}\right)}$
		eCT3nw		${s}_{eCT3\left(1s\_d\right)}=\frac{\mathrm{ln}\left(1+\sum_{1-s}{f}_{s}\left({\Delta }_{n(k)}\right){C}_{n(k)}\right)}{\mathrm{ln}\left(1+\sum_{s}{C}_{n(k)}+\sum_{d}{C}_{n(k)}\right)}$
	eCT3_0	eCT30w		${s}_{eCT3\left(s\_wd\right)}=\frac{\mathrm{ln}\left(1+\sum_{s}{f}_{s}\left({\Delta }_{n(k)}\right){C}_{n(k)}\right)}{\mathrm{ln}\left(1+\sum_{s}{f}_{s}\left({\Delta }_{n(k)}\right){C}_{n(k)}+\sum_{d}{f}_{d}\left({\Delta }_{n(k)}\right){C}_{n(k)}\right)}$
		eCT30nw		${s}_{eCT3\left(s\_d\right)}=\frac{\mathrm{ln}\left(1+\sum_{s}{f}_{s}\left({\Delta }_{n(k)}\right){C}_{n(k)}\right)}{\mathrm{ln}\left(1+\sum_{s}{C}_{n(k)}+\sum_{d}{C}_{n(k)}\right)}$
eCT4	eCT4_1	eCT4w	Extended Consonni-Todeschini (4)	${s}_{eCT4\left(1s\_wd\right)}=\frac{\mathrm{ln}\left(1+\sum_{1-s}{f}_{s}\left({\Delta }_{n(k)}\right){C}_{n(k)}\right)}{\mathrm{ln}\left(1+\sum_{1-s}{f}_{s}\left({\Delta }_{n(k)}\right){C}_{n(k)}+\sum_{d}{f}_{d}\left({\Delta }_{n(k)}\right){C}_{n(k)}\right)}$
		eCT4nw		${s}_{eCT4\left(1s\_d\right)}=\frac{\mathrm{ln}\left(1+\sum_{1-s}{f}_{s}\left({\Delta }_{n(k)}\right){C}_{n(k)}\right)}{\mathrm{ln}\left(1+\sum_{1-s}{C}_{n(k)}+\sum_{d}{C}_{n(k)}\right)}$
	eCT4_0	eCT40w		${s}_{eCT4\left(s\_wd\right)}=\frac{\mathrm{ln}\left(1+\sum_{s}{f}_{s}\left({\Delta }_{n(k)}\right){C}_{n(k)}\right)}{\mathrm{ln}\left(1+\sum_{s}{f}_{s}\left({\Delta }_{n(k)}\right){C}_{n(k)}+\sum_{d}{f}_{d}\left({\Delta }_{n(k)}\right){C}_{n(k)}\right)}$
		eCT4nw		${s}_{eCT4\left(s\_d\right)}=\frac{\mathrm{ln}\left(1+\sum_{s}{f}_{s}\left({\Delta }_{n(k)}\right){C}_{n(k)}\right)}{\mathrm{ln}\left(1+\sum_{s}{C}_{n(k)}+\sum_{d}{C}_{n(k)}\right)}$
eGle	eGle_1	eGlew	Extended Gleason	${s}_{eGle\left(1s\_wd\right)}=\frac{2\sum_{1-s}{f}_{s}\left({\Delta }_{n(k)}\right){C}_{n(k)}}{2\sum_{1-s}{f}_{s}\left({\Delta }_{n(k)}\right){C}_{n(k)}+\sum_{d}{f}_{d}\left({\Delta }_{n(k)}\right){C}_{n(k)}}$
		eGlenw		${s}_{eGle\left(1s\_d\right)}=\frac{2\sum_{1-s}{f}_{s}\left({\Delta }_{n(k)}\right){C}_{n(k)}}{2\sum_{1-s}{C}_{n(k)}+\sum_{d}{C}_{n(k)}}$
	eGle_0	eGle0w		${s}_{eGle\left(s\_wd\right)}=\frac{2\sum_{s}{f}_{s}\left({\Delta }_{n(k)}\right){C}_{n(k)}}{2\sum_{s}{f}_{s}\left({\Delta }_{n(k)}\right){C}_{n(k)}+\sum_{d}{f}_{d}\left({\Delta }_{n(k)}\right){C}_{n(k)}}$
		eGle0nw		${s}_{eGle\left(s\_d\right)}=\frac{2\sum_{s}{f}_{s}\left({\Delta }_{n(k)}\right){C}_{n(k)}}{2\sum_{s}{C}_{n(k)}+\sum_{d}{C}_{n(k)}}$
eJa	eJa_1	eJaw	Extended Jaccard	${s}_{eJa\left(1s\_wd\right)}=\frac{3\sum_{1-s}{f}_{s}\left({\Delta }_{n(k)}\right){C}_{n(k)}}{3\sum_{1-s}{f}_{s}\left({\Delta }_{n(k)}\right){C}_{n(k)}+\sum_{d}{f}_{d}\left({\Delta }_{n(k)}\right){C}_{n(k)}}$
		eJanw		${s}_{eJa\left(1s\_d\right)}=\frac{3\sum_{1-s}{f}_{s}\left({\Delta }_{n(k)}\right){C}_{n(k)}}{3\sum_{1-s}{C}_{n(k)}+\sum_{d}{C}_{n(k)}}$
	eJa_0	eJa0w		${s}_{eJa\left(s\_wd\right)}=\frac{3\sum_{s}{f}_{s}\left({\Delta }_{n(k)}\right){C}_{n(k)}}{3\sum_{s}{f}_{s}\left({\Delta }_{n(k)}\right){C}_{n(k)}+\sum_{d}{f}_{d}\left({\Delta }_{n(k)}\right){C}_{n(k)}}$
		eJa0nw		${s}_{eJa\left(s\_d\right)}=\frac{3\sum_{s}{f}_{s}\left({\Delta }_{n(k)}\right){C}_{n(k)}}{3\sum_{s}{C}_{n(k)}+\sum_{d}{C}_{n(k)}}$
eRR	eRR_1	eRRw	Extended Russel-Rao	${s}_{eRR\left(1s\_wd\right)}=\frac{\sum_{1-s}{f}_{s}\left({\Delta }_{n(k)}\right){C}_{n(k)}}{\sum_{s}{f}_{s}\left({\Delta }_{n(k)}\right){C}_{n(k)}+\sum_{d}{f}_{d}\left({\Delta }_{n(k)}\right){C}_{n(k)}}$
		eRRnw		${s}_{eRR\left(1s\_d\right)}=\frac{\sum_{1-s}{f}_{s}\left({\Delta }_{n(k)}\right){C}_{n(k)}}{\sum_{s}{C}_{n(k)}+\sum_{d}{C}_{n(k)}}$
	eRR_0	eRR0w		${s}_{eRR\left(s\_wd\right)}=\frac{\sum_{s}{f}_{s}\left({\Delta }_{n(k)}\right){C}_{n(k)}}{\sum_{s}{f}_{s}\left({\Delta }_{n(k)}\right){C}_{n(k)}+\sum_{d}{f}_{d}\left({\Delta }_{n(k)}\right){C}_{n(k)}}$
		eRR0nw		${s}_{eRR\left(s\_d\right)}=\frac{\sum_{s}{f}_{s}\left({\Delta }_{n(k)}\right){C}_{n(k)}}{\sum_{s}{C}_{n(k)}+\sum_{d}{C}_{n(k)}}$
eSS1	eSS1_0	eSSw	Extended Sokal-Sneath (1)	${s}_{eSS1\left(1s\_wd\right)}=\frac{\sum_{1-s}{f}_{s}\left({\Delta }_{n(k)}\right){C}_{n(k)}}{\sum_{1-s}{f}_{s}\left({\Delta }_{n(k)}\right){C}_{n(k)}+2\sum_{d}{f}_{d}\left({\Delta }_{n(k)}\right){C}_{n(k)}}$
		eSSnw		${s}_{eSS1\left(1s\_d\right)}=\frac{\sum_{1-s}{f}_{s}\left({\Delta }_{n(k)}\right){C}_{n(k)}}{\sum_{1-s}{C}_{n(k)}+2\sum_{d}{C}_{n(k)}}$
	eSS1_1	eSS0w		${s}_{eSS1\left(s\_wd\right)}=\frac{\sum_{s}{f}_{s}\left({\Delta }_{n(k)}\right){C}_{n(k)}}{\sum_{s}{f}_{s}\left({\Delta }_{n(k)}\right){C}_{n(k)}+2\sum_{d}{f}_{d}\left({\Delta }_{n(k)}\right){C}_{n(k)}}$
		eSS0nw		${s}_{eSS1\left(s\_d\right)}=\frac{\sum_{s}{f}_{s}\left({\Delta }_{n(k)}\right){C}_{n(k)}}{\sum_{s}{C}_{n(k)}+2\sum_{d}{C}_{n(k)}}$
eJT	eJT_1	eJTw	Extended Jaccard-Tanimoto	${s}_{eJT\left(1s\_wd\right)}=\frac{\sum_{1-s}{f}_{s}\left({\Delta }_{n(k)}\right){C}_{n(k)}}{\sum_{1-s}{f}_{s}\left({\Delta }_{n(k)}\right){C}_{n(k)}+\sum_{d}{f}_{d}\left({\Delta }_{n(k)}\right){C}_{n(k)}}$
		eJTnw		${s}_{eJT\left(1s\_d\right)}=\frac{\sum_{1-s}{f}_{s}\left({\Delta }_{n(k)}\right){C}_{n(k)}}{\sum_{1-s}{C}_{n(k)}+\sum_{d}{C}_{n(k)}}$
	eJT_0	eJT0w		${s}_{eJT\left(s\_wd\right)}=\frac{\sum_{s}{f}_{s}\left({\Delta }_{n(k)}\right){C}_{n(k)}}{\sum_{s}{f}_{s}\left({\Delta }_{n(k)}\right){C}_{n(k)}+\sum_{d}{f}_{d}\left({\Delta }_{n(k)}\right){C}_{n(k)}}$
		eJT0nw		${s}_{eJT\left(s\_d\right)}=\frac{\sum_{s}{f}_{s}\left({\Delta }_{n(k)}\right){C}_{n(k)}}{\sum_{s}{C}_{n(k)}+\sum_{d}{C}_{n(k)}}$
