# Supplementary material for: Extended similarity indices: the benefits of comparing more than two objects simultaneously. Part 2: speed, consistency, diversity selection
Source: J Cheminform. 2021 Apr 23;13:33. doi: 10.1186/s13321-021-00504-4 (PMC8067665; doi:10.1186/s13321-021-00504-4)
Supplement: Supplementary file 1 — Additional file 1: Figure S1: Average time required to calculate the set similarity of the different datasets using Morgan4 fingerprints with binary similarity indices. Figure S2: Average time required to calculate the set similarity of the different datasets using Morgan4 fingerprints with n-ary similarity indices. Table S1: Average internal consistency fractions over sets with 10, 20, …, 300 molecules of all the extended similarity indices for all fingerprint types. Table S2: Average external consistency fractions over sets with 10, 20,…, 300 molecules of all the extended similarity indices for all fingerprint types. Figure S3: SRD analysis for the internal (i) and external (e) consistencies over the different fingerprint types. Figure S4: Effect of internal (i) and external consistency (e) on the extended multiple similarity indices. Notation can be found in Appendix 1, and also in the accompanying paper.4. Figure S5: Effect of weighting on the extended multiple similarity indices. Figure S6: Joint effect of internal and external consistency as well as weighting on the extended multiple similarity indices. [file 13321_2021_504_MOESM1_ESM.docx]

**Supplementary material**

**Extended similarity indices: the benefits of comparing more than two objects simultaneously. Part 2: Speed, consistency, diversity selection**

Ramón Alain Miranda-Quintana^1^, Dávid Bajusz^2^, Anita Rácz^3^, Károly Héberger^3^

*^1^ Department of Chemistry, University of Florida, Gainesville, FL 32603, USA*

*^2^ Medicinal Chemistry Research Group, Research Centre for Natural Sciences, Magyar tudósok krt. 2, 1117, Budapest, Hungary*

*^3^ Plasma Chemistry Research Group, Research Centre for Natural Sciences, Magyar tudósok krt. 2, 1117, Budapest, Hungary*

**Section 1: Time analysis**

**Figure S1:** Average time required to calculate the set similarity of the different datasets using Morgan4 fingerprints with *binary* similarity indices.

|  |
| --- |
|  |
|  |

**Figure S2:** Average time required to calculate the set similarity of the different datasets using Morgan4 fingerprints with *n-ary* similarity indices.

|  |
| --- |
|  |
|  |

The comparison of figures S1 and S2 illustrates the drastic difference in computation times in favor of multiple comparisons.

**Section 2: Internal consistency analysis**

In Table S1 we present the internal consistency fraction (ICF) for each of the different fingerprint types. For all the given fingerprints and indices, the value reported corresponds to the ICF averaged over the sets with *n* = 10, 100, 1000, 10000, 100000. In this way, columns 2-5 give a measure of how the internal consistency of and index depends on the fingerprint type. In a similar way, the last column in Table 1 averages the ICF over all fingerprint types, giving a coarse measure of the internal consistency of an index. The first conclusion is that weighting of an index tends to decrease its internal consistency. This tendency is observed in almost all cases, with most of the exceptions appearing when we use the Morgan4_4096 fingerprints.

**Table S1:** Average internal consistency fractions over sets with 10, 20, …, 300 molecules of all the extended similarity indices for all fingerprint types. ICF ≥ 0.9 values highlighted in green.

| Extended indices | MACSS | Morgan4  1024 | Morgan4  2048 | Morgan4  4096 | Average |
| --- | --- | --- | --- | --- | --- |
| eACnw | **1.00** | **0.94** | **0.96** | **0.96** | **0.97** |
| eACw | 0.84 | **0.92** | 0.86 | 0.87 | 0.87 |
| eBUBnw | **0.96** | **0.90** | **0.90** | **0.90** | **0.91** |
| eBUBw | 0.81 | 0.82 | 0.77 | 0.76 | 0.79 |
| eCT1nw | **1.00** | **0.94** | **0.96** | **0.96** | **0.97** |
| eCT1w | 0.84 | **0.92** | 0.86 | 0.87 | 0.87 |
| eCT2nw | 0.87 | **0.95** | **0.93** | **0.94** | **0.92** |
| eCT2w | 0.84 | **0.91** | 0.86 | 0.87 | 0.87 |
| eCT3nw | **0.91** | **0.99** | **0.98** | **0.98** | **0.96** |
| eCT3w | **0.92** | **1.00** | **1.00** | **0.98** | **0.97** |
| eCT4nw | **0.97** | 0.81 | 0.82 | 0.82 | 0.86 |
| eCTw | 0.87 | 0.81 | 0.85 | 0.83 | 0.84 |
| eFainw | **1.00** | **0.93** | **0.93** | **0.90** | **0.94** |
| eFaiw | 0.81 | 0.78 | 0.74 | 0.76 | 0.77 |
| eGKnw | **0.96** | 0.63 | 0.63 | 0.63 | 0.71 |
| eGKw | 0.82 | 0.67 | 0.67 | 0.60 | 0.69 |
| eGlenw | **0.96** | 0.86 | 0.77 | 0.79 | 0.85 |
| eGlew | 0.83 | 0.78 | 0.79 | 0.82 | 0.80 |
| eHDnw | **0.95** | **0.97** | 0.80 | 0.79 | 0.88 |
| eHDw | 0.81 | 0.80 | 0.77 | 0.80 | 0.79 |
| eJTnw | **0.97** | 0.84 | 0.76 | 0.77 | 0.84 |
| eJTw | 0.83 | 0.78 | 0.79 | 0.82 | 0.80 |
| eJanw | **0.97** | 0.88 | 0.79 | 0.82 | 0.87 |
| eJaw | 0.83 | 0.78 | 0.79 | 0.82 | 0.80 |
| eJa0nw | **1.00** | **0.94** | **0.96** | **0.96** | **0.97** |
| eJa0w | 0.84 | 0.92 | 0.86 | 0.87 | 0.87 |
| eRGnw | **0.99** | **0.95** | 0.83 | 0.83 | **0.90** |
| eRGw | 0.81 | 0.78 | 0.78 | 0.82 | 0.80 |
| eRRnw | **0.91** | **0.99** | **0.98** | **0.98** | **0.96** |
| eRRw | **0.93** | **1.00** | **1.00** | **1.00** | **0.98** |
| eRTnw | **0.93** | **0.94** | **0.96** | **0.96** | **0.95** |
| eRTw | 0.84 | **0.92** | 0.86 | 0.87 | 0.87 |
| eSMnw | **1.00** | **0.94** | **0.96** | **0.96** | **0.97** |
| eSMw | 0.84 | **0.92** | 0.86 | 0.87 | 0.87 |
| eSSnw | **0.97** | 0.80 | 0.75 | 0.73 | 0.81 |
| eSSw | 0.83 | 0.78 | 0.79 | 0.82 | 0.80 |
| eSS2nw | **1.00** | **0.94** | **0.96** | **0.96** | **0.97** |
| eSS2w | 0.84 | **0.92** | 0.86 | 0.87 | 0.87 |

The results in Table S1 also highlight which fingerprints contribute to preserving the internal consistency of the similarity indices. For instance, when we work with MACSS fingerprints 20 of the 38 extended indices have an ICF ≥ 0.9, while all the indices have an ICF of at least 0.8. Comparably, using the Morgan4_1024 fingerprints leads to 21 indices with ICF ≥ 0.9 (but only 28 with ICF ≥ 0.8). On the other hand, when we use Morgan4_2048 or Morgan4_4096 fingerprints there are only 12 indices with ICF ≥ 0.9 (although in both cases there are at least 24 indices with ICF ≥ 0.8). This clearly shows how *the choice of fingerprint type can impact the results of a comparative analysis. This feature might be used for the selection of better fingerprints than used currently*.

Finally, if we look at the overall ICF values reported in the last column of Table 1 we see that most of the indices (32 out of 38) have an ICF ≥ 0.8, with 14 having ICF ≥ 0.9. So, with the exception of some pathologically ill-behaved cases (like the eGK index), the rest of the indices present a great degree of internal consistency. On the other hand, indices like the non-weighted eACnw, eCT1nw, eCT3nw, eJanw, eRRnw, eSMnw, and eSS2nw present a remarkable internal consistency, with ICF values of at least 0.96. The best weighted and non-weighted versions of the same index were the eCT3 and eRR indices, which in all cases have an ICF ≥ 0.96 (the biggest ICF value was 0.98, for the weighted eRRw index). Before concluding this section, it is reassuring to note that the two indices that we identified as the best thanks to the sum of ranking differences (SRD, a multiobject optimization tool, see Part 1) in our previous work (the eBUBnw and eFainw indices) have an ICF ≥ 0.9 when they are not weighted.^1^

**Section 3: External consistency analysis**

Here we also report the external consistency fractions (ECF) for all the studied indices and fingerprints. To be as representative and general as possible, the values in Table S2 were obtained in the following way: given an *n*-ary index and a fingerprint type, we calculated the ECF for all the datasets with 10, 20, …, 290, 300 molecules over all the possible coincidence thresholds, we then averaged those numbers so as to have a measure of the external consistency of the index for the selected fingerprint type.

**Table S2:** Average external consistency fractions over sets with 10, 20,…, 300 molecules of all the extended similarity indices for all fingerprint types. Also shown: average over all fingerprint types, and average over the Morgan4 fingerprints. ECF ≥ 0.9 values highlighted in green.

| Extended indices | MACSS | Morgan4  1024 | Morgan4  2048 | Morgan4  4096 | Average  All | Average  Morgan4 |
| --- | --- | --- | --- | --- | --- | --- |
| eACnw | 0.79 | 0.59 | 0.55 | 0.51 | 0.61 | 0.55 |
| eACw | 0.62 | 0.11 | 0.06 | 0.05 | 0.21 | 0.07 |
| eBUBnw | 0.76 | 0.87 | 0.81 | 0.77 | 0.80 | 0.82 |
| eBUBw | 0.65 | 0.45 | 0.53 | 0.41 | 0.51 | 0.47 |
| eCT1nw | 0.79 | 0.59 | 0.55 | 0.51 | 0.61 | 0.55 |
| eCT1w | 0.63 | 0.10 | 0.06 | 0.05 | 0.21 | 0.07 |
| eCT2nw | 0.62 | 0.11 | 0.07 | 0.06 | 0.21 | 0.08 |
| eCT2w | 0.58 | 0.11 | 0.06 | 0.05 | 0.20 | 0.07 |
| eCT3nw | 0.53 | 0.03 | 0.10 | 0.25 | 0.23 | 0.13 |
| eCT3w | 0.53 | 0.03 | 0.10 | 0.25 | 0.23 | 0.13 |
| eCT4nw | **0.93** | **0.95** | **0.95** | **0.95** | **0.94** | **0.95** |
| eCTw | 0.85 | 0.82 | 0.85 | 0.85 | 0.84 | 0.84 |
| eFainw | **1.00** | 0.60 | 0.37 | 0.29 | 0.56 | 0.42 |
| eFaiw | **0.99** | 0.37 | 0.31 | 0.29 | 0.49 | 0.32 |
| eGKnw | 0.14 | 0.12 | 0.12 | 0.11 | 0.12 | 0.11 |
| eGKw | 0.13 | 0.18 | 0.15 | 0.13 | 0.15 | 0.16 |
| eGlenw | 0.86 | **0.95** | **0.94** | **0.93** | **0.92** | **0.94** |
| eGlew | 0.80 | 0.82 | 0.85 | 0.84 | 0.83 | 0.84 |
| eHDnw | 0.70 | 0.74 | 0.81 | 0.85 | 0.77 | 0.80 |
| eHDw | 0.62 | 0.73 | 0.81 | 0.84 | 0.75 | 0.79 |
| eJTnw | 0.86 | **0.95** | **0.94** | **0.93** | **0.92** | **0.94** |
| eJTw | 0.80 | 0.82 | 0.85 | 0.84 | 0.83 | 0.84 |
| eJanw | 0.86 | **0.94** | **0.94** | **0.94** | **0.92** | **0.94** |
| eJaw | 0.80 | 0.82 | 0.85 | 0.84 | 0.83 | 0.84 |
| eJa0nw | 0.72 | **0.94** | **0.96** | **0.95** | 0.89 | **0.95** |
| eJa0w | 0.62 | 0.11 | 0.06 | 0.05 | 0.21 | 0.07 |
| eRGnw | 0.75 | 0.79 | 0.86 | **0.90** | 0.82 | 0.85 |
| eRGw | 0.65 | 0.77 | 0.84 | 0.84 | 0.78 | 0.82 |
| eRRnw | 0.17 | 0.03 | 0.10 | 0.13 | 0.11 | 0.09 |
| eRRw | 0.19 | 0.04 | 0.10 | 0.13 | 0.11 | 0.09 |
| eRTnw | 0.74 | 0.39 | 0.39 | 0.36 | 0.47 | 0.38 |
| eRTw | 0.62 | 0.11 | 0.06 | 0.05 | 0.21 | 0.07 |
| eSMnw | 0.79 | 0.59 | 0.55 | 0.51 | 0.61 | 0.55 |
| eSMw | 0.62 | 0.11 | 0.06 | 0.05 | 0.21 | 0.07 |
| eSSnw | 0.85 | **0.93** | **0.92** | **0.91** | **0.90** | **0.92** |
| eSSw | 0.80 | 0.82 | 0.85 | 0.84 | 0.83 | 0.84 |
| eSS2nw | 0.84 | **0.91** | 0.87 | 0.82 | 0.86 | 0.87 |
| eSS2w | 0.62 | 0.11 | 0.06 | 0.05 | 0.21 | 0.07 |

As it was the case for the internal consistency, overall the non-weighted indices tend to be more consistent than their weighted counterparts. We also confirm that there is a close interplay between the fingerprint type and the consistency results. Notice how the eFainw index shows an ECF of 1 when we use MACSS fingerprints, which means that in this case this index is externally consistent over all datasets and coincidence thresholds (which is truly remarkable). But, as soon as we move to the Morgan4 fingerprints the external consistency of the eFainw index rapidly deteriorates, with rather low ECF values always below 0.6. These differences appear in other cases, most notably in the weighted versions of the eAC and eCT1 indices, denoted by eACw and eCT1w , and for both variants of the eCT2 and eCT3 indices. On the other hand, for many indices the overall performance is relatively the same for all the fingerprint types (*e.g*., see the eJT and eRG indices). In general, when we take the average over all fingerprint types (6^th^ column in Table 2), we see that there are five indices with ECF ≥ 0.9: eCT4nw (with an overall best of 0.94), eGlenw, eJTnw, eJanw, and eSS1nw (all in their non-weighted versions). If we only take the average over the Morgan4 fingerprints, then also the non-weighted eJa0nw index has an ECF ≥ 0.9. It is once again reassuring to see that the non-weighted eBUBnw index has an adequate ECF of 0.8, which reaffirms its generally good performance over all the indicators considered. The Baroni-Urbani-Buser index proved to be (one of) the best in a metabolomics application.[^1^](#_ENREF_49) Larger scale comparisons of 2D fingerprints and similarity indices also support the advantageous usage of the BUB index during the complex optimization task.[^2^](#_ENREF_50)

**Section 4: Comparison of fingerprints and extended similarity indices**

The data of tables S1 and S2 are also suitable to make a multicriteria decision analysis as described in our earlier publication^3^ using sum of ranking differences and coupled to ANOVA.

First the comparison of fingerprints type was completed. The methodology uses sum of ranking differences and variance analysis as described in Part 1, where the maximum has been chosen as gold standard (reference). The corresponding results are presented in Figure S3.


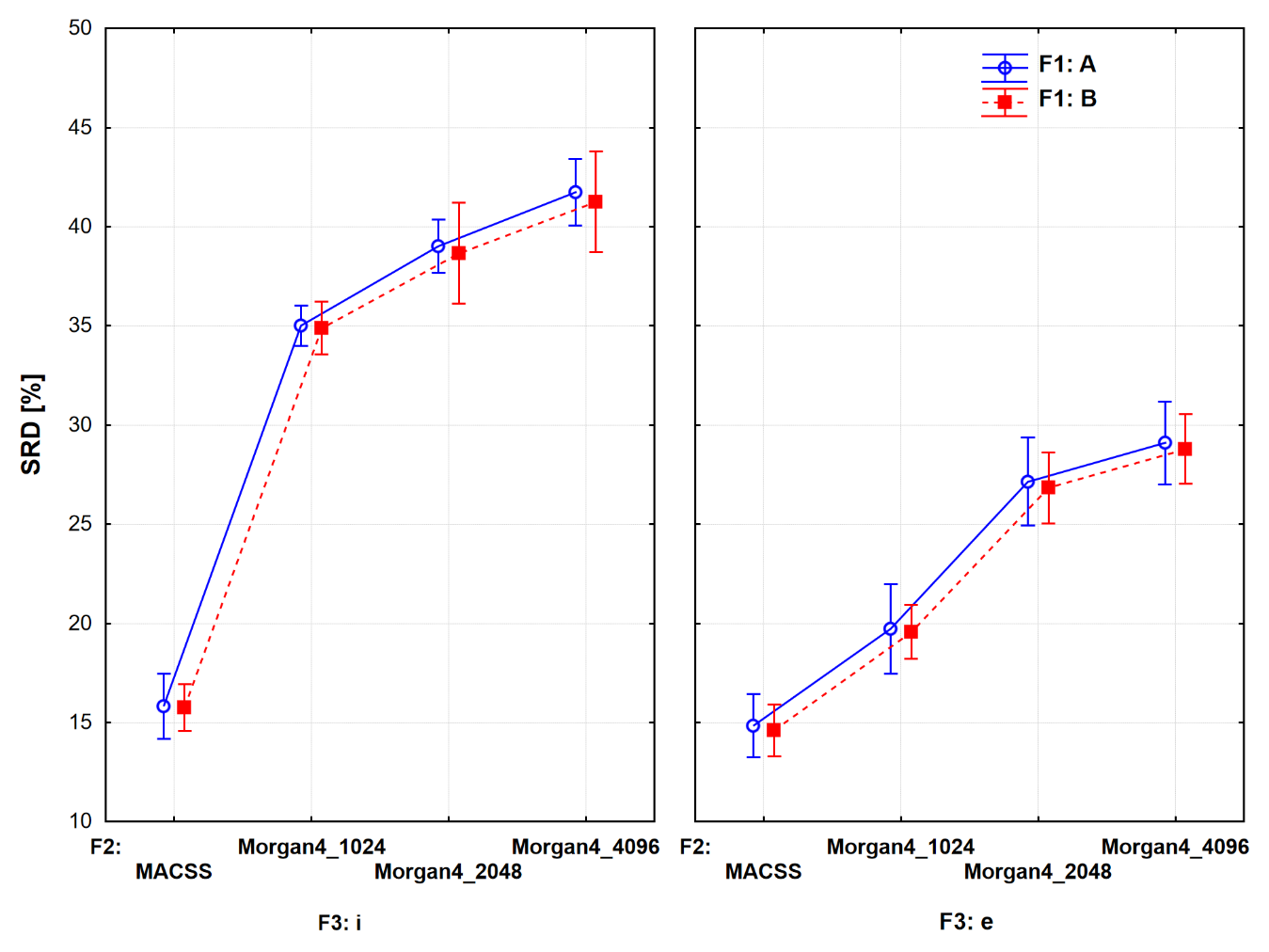


**Figure S3:** SRD analysis for the internal (i) and external (e) consistencies over the different fingerprint types.

A and B represent the cross-validation variant (A–contiguous, B–random resampling with return). This factor is not significant, but this is reassuring, no error was introduced during cross-validation. Whereas the internal and external consistency does not differ much for MACCS fingerprint, the external consistency for Morgan fingerprints is more advantageous (smaller SRD is closer to the reference, in this case the maximum external consistency.)

Second, we transposed the input matrix composed form data of Tables S1 and S2. The next three figures show the interplay of various factors.


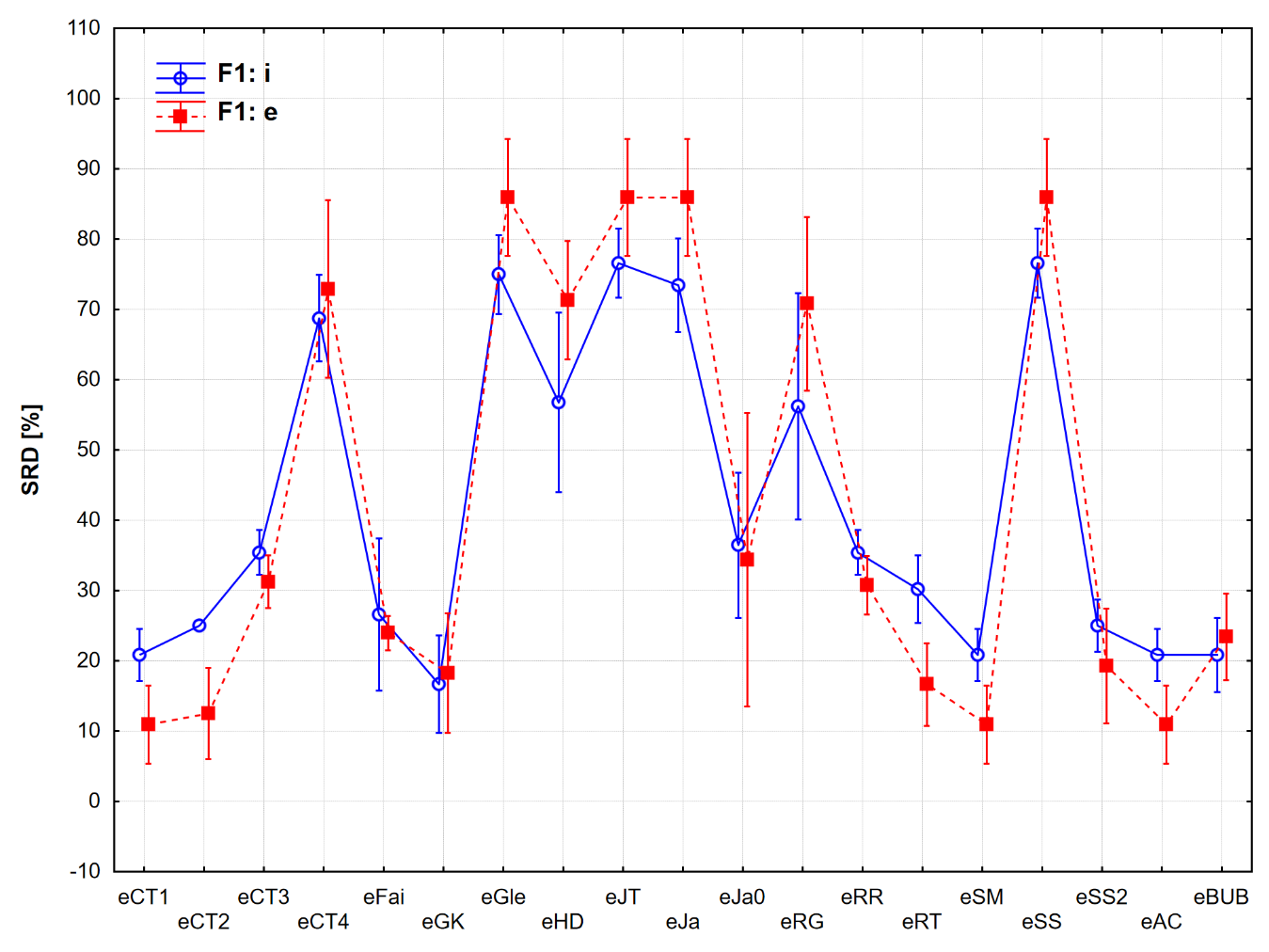


**Figure S4:** Effect of internal (i) and external consistency (e) on the extended multiple similarity indices. Notation can be found in Appendix 1, and also in the accompanying paper.^4^

It is remarkable that, overall, we observe the same general trends, that is, indices that tend to be more internally consistent also tend to be more externally consistent (Figure 8). It is also interesting to see the different relative importance of external and internal consistency for a given index. In this case we observe different behaviors: in some cases, the external consistency is predominant, *e.g*. for eCT1, eCT2, eRT, eSM, eAC, it is in level with the internal consistency for eFai, eGK, eJa0, and eBUB, while eGle, eHD, eJT, eRG tend to be more internally than externally consistent.


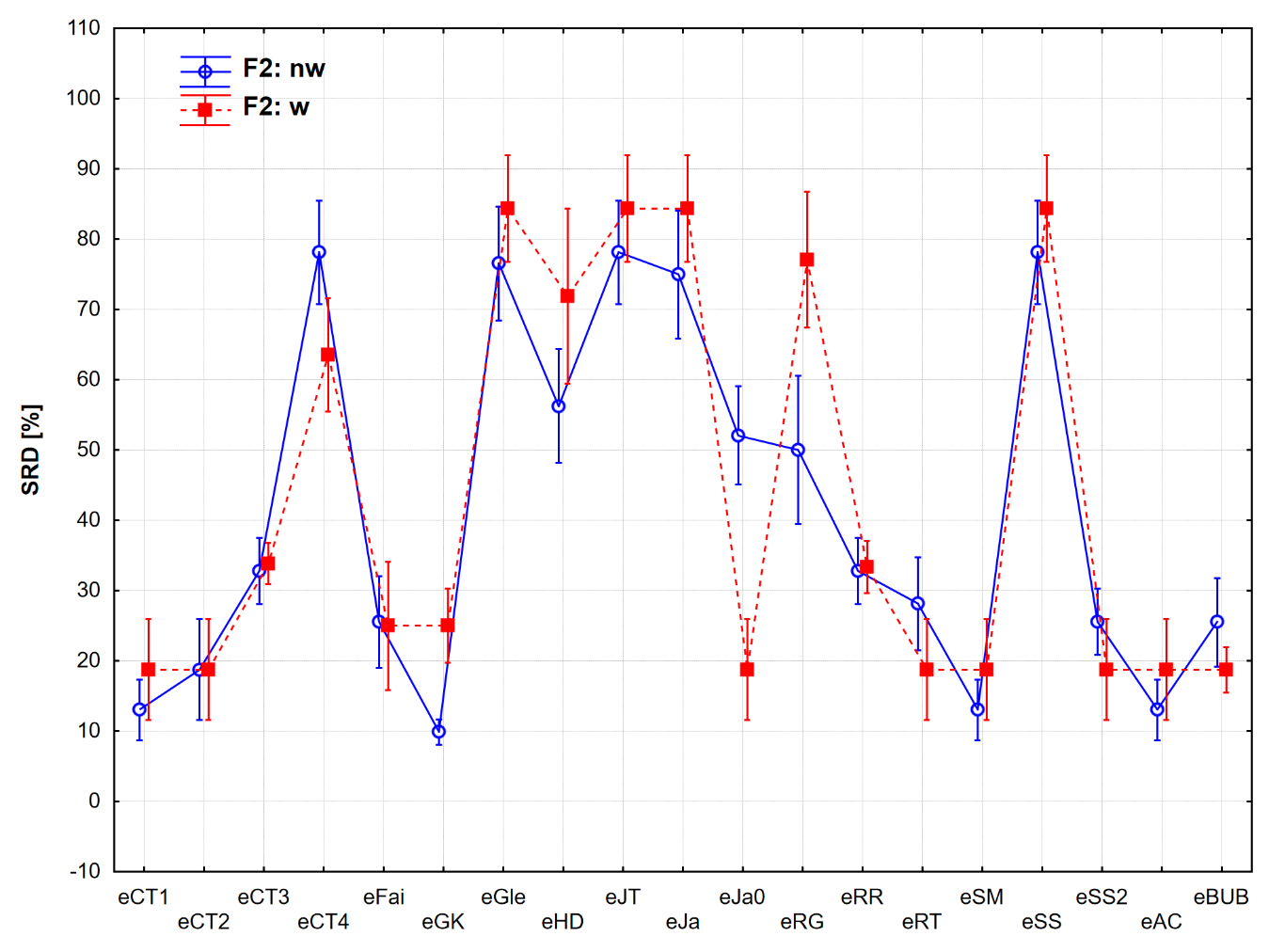


**Figure S5:** Effect of weighting on the extended multiple similarity indices (w-weighted (red), nw-non-weighted (blue).

The effect of weighting on the consistency types also shows the same overall trend for the studied indices. Interestingly, weighting noticeably improves the performance of eJa0, which is also the index that shows the largest discrepancy between weighted and non-weighted indices (Figure S5).


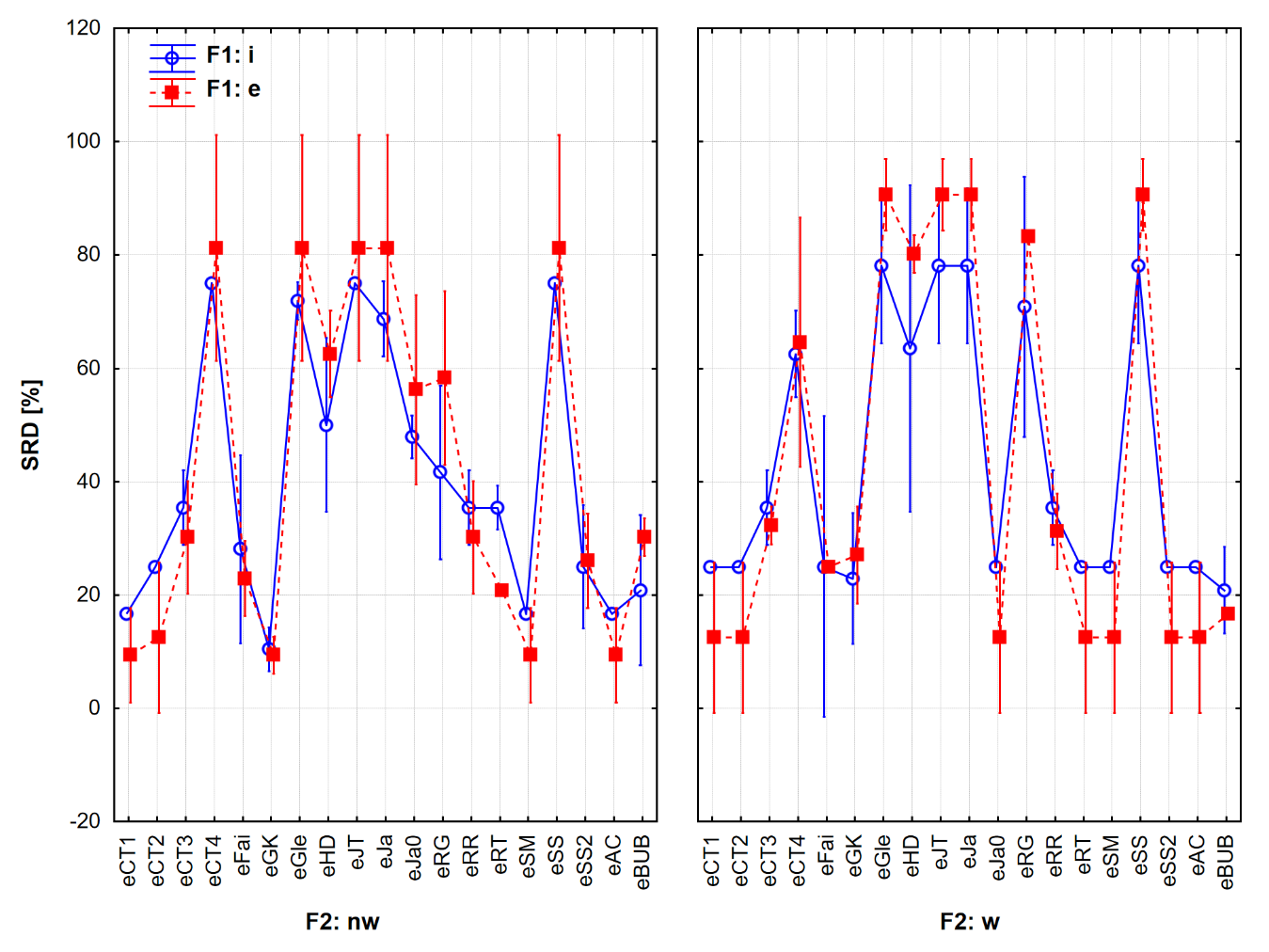


**Figure S6:** Joint effect of internal and external consistency as well as weighting on the extended multiple similarity indices.

The interplay between the consistency types and the weighting is shown in Figure S6, where once again we see that overall the indices follow the same general trends. Here we can see SRD scores around 80 are overlapping with the random ranking. On the other hand, the indices with smallest SRD scores can be recommended for further usage (notice that eJa0 is only suitable in its weighted form).

**References**

(1) Anita Rácz, Filip Andrić, Dávid Bajusz, Károly Héberger, Binary similarity measures for fingerprint analysis of qualitative metabolomic profiles, Metabolomics, 14, Article Number: 29. pp. 1-9 (2018) https://doi.org/10.1007/s11306-018-1327-y.

(2) Naomie Salim, John Holliday, and Peter Willett, Combination of Fingerprint-Based Similarity Coefficients Using Data Fusion, J. Chem. Inf. Comput. Sci. 43, 435-442 (2003) <https://doi.org/10.1021/ci025596j>

(3) Dávid Bajusz, Anita Rácz, Károly Héberger, Why is Tanimoto index an appropriate choice for fingerprint-based similarity calculations? Journal of Cheminformatics, 7, 20 (2015). https://doi.org/10.1186/s13321-015-0069-3

(4) Ramón Alain Miranda-Quintana, Dávid Bajusz, Anita Rácz, Károly Héberger, Extended similarity indices: the benefits of comparing more than two objects simultaneously. Part 1: Theory and characteristics, J. Cheminform. (2021) <https://doi.org/10.1186/s13321-021-00505-3>
